# Development of Urea-Bond-Containing Michael Acceptors as Antitrypanosomal Agents Targeting Rhodesain

**DOI:** 10.1021/acsmedchemlett.2c00084

**Published:** 2022-06-30

**Authors:** Santo Previti, Roberta Ettari, Elsa Calcaterra, Carla Di Chio, Rahul Ravichandran, Collin Zimmer, Stefan Hammerschmidt, Annika Wagner, Marta Bogacz, Sandro Cosconati, Tanja Schirmeister, Maria Zappalà

**Affiliations:** † Department of Chemical, Biological, Pharmaceutical and Environmental Sciences, University of Messina, Viale Ferdinando Stagno d’Alcontres 31, 98166 Messina, Italy; ‡ DiSTABiF, University of Campania “Luigi Vanvitelli”, Via Vivaldi 43, 81100 Caserta, Italy; § Institute of Pharmaceutical and Biomedical Sciences, University of Mainz, Staudingerweg 5, 55128 Mainz, Germany; ∥ Institute of Organic Chemistry & Macromolecular Chemistry, Friedrich-Schiller-University of Jena, Humboldtstraße 10, 07743 Jena, Germany

**Keywords:** rhodesain, sleeping sickness, Michael acceptors, urea bond, antitrypanosomal

## Abstract

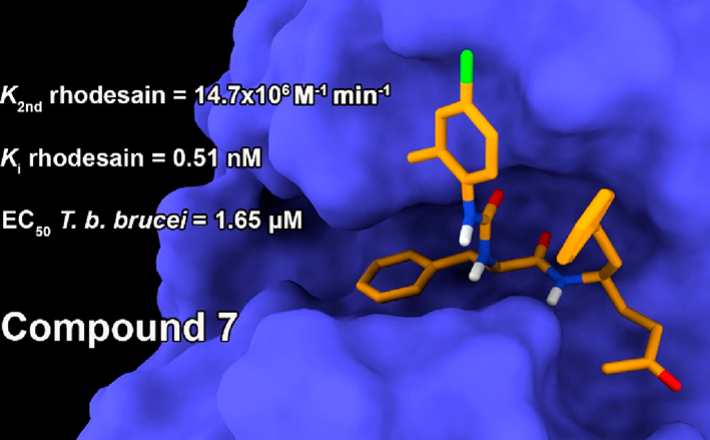

Human African Trypanosomiasis (HAT) is a neglected tropical disease widespread in sub-Saharan Africa. Rhodesain, a cysteine protease of *Trypanosoma brucei rhodesiense*, has been identified as a valid target for the development of anti-HAT agents. Herein, we report a series of urea-bond-containing Michael acceptors, which were demonstrated to be potent rhodesain inhibitors with *K*_i_ values ranging from 0.15 to 2.51 nM, and five of them showed comparable *k*_2nd_ values to that of K11777, a potent antitrypanosomal agent. Moreover, most of the urea derivatives exhibited single-digit micromolar activity against the protozoa, and the presence of substituents at the P3 position appears to be essential for the antitrypanosomal effect. Replacement of Phe with Leu at the P2 site kept unchanged the inhibitory properties. Compound **7** (SPR7) showed the best compromise in terms of rhodesain inhibition, selectivity, and antiparasitic activity, thus representing a new lead compound for future SAR studies.

Human African Trypanosomiasis, also known by its acronym HAT or sleeping sickness, is a vector-borne Neglected Tropical Disease (NTD) caused by protozoa of *Trypanosoma brucei* (*T. b.*) species, widespread in sub-Saharan Africa.^1,2^ Two morphologically identical subspecies cause a different progression of the infection: *T. b. gambiense* is responsible for the chronic form of HAT characterized by slow development (*g*HAT); meanwhile, *T. b. rhodesiense* causes the acute form (*r*HAT).^2^ The clinical manifestations of the disease were classified into two stages, namely, the hemolymphatic and neurological stages (stages 1 and 2, respectively).^3,4^ During stage 1, the protozoa, inoculated by the tsetse fly bite, mainly remain in the hemolymphatic system, spleen, and interstitial spaces, resulting in nonspecific symptoms, such as fever and general malaise.^5,6^ Subsequently, for still unclear reasons, the protozoa cross the blood-brain barrier (BBB) and invade the central nervous system (CNS), giving rise to stage 2, which is characterized by severe neurological problems, such as mental confusion, delirium, coma, and, last, death.^7,8^ As mentioned above, the two forms of HAT deeply differ in the disease manifestation progression: in fact, while *g*HAT can last for years, *r*HAT is characterized by a rapid progression (4–5 weeks) and higher mortality rate.^9^ To date, chemotherapy is the sole strategy to treat the infection: suramin, pentamidine, melarsoprol, and eflornithine were largely employed in the last century with modest results, mainly due to their toxicity, narrow-spectrum activity, and onset of resistance.^10,11^ In the last decades, nifurtimox, a well-known nitrofuran used to treat Chagas disease, was off-label employed in combination with eflornithine for the treatment of the neurological stage of *g*HAT.^11^ More recently, the nitroimidazole derivative fexinidazole was approved by the U.S. Food and Drug Administration (FDA) for the treatment of both stages of *g*HAT.^12,13^ At present, the enormous efforts made by the World Health Organization (WHO), Drugs for Neglected Diseases Initiative (DNDi), and charitable foundations have led to a significant decrease in HAT cases and related deaths.^14^ Despite that, the data collected could be underestimated due to the difficulty in reaching the most remote African regions. Furthermore, the possibility to easily move worldwide could spread the disease in nonendemic countries.^15,16^ Last but not least, both Nifurtimox-Eflornithine Combination Therapy (NECT) and fexinidazole were approved against *g*HAT, while the most aggressive and lethal *r*HAT does not have a drug of choice.^11,12^

Starting from these considerations, the efforts of medicinal chemists were focused on the identification of novel approaches for HAT treatment. For this purpose, several review articles report innovative and valid strategies useful for the identification of novel targets and antitrypanosomal agents.^17−19^ In this context, rhodesain, a cysteine protease of *T. b. rhodesiense*, turned out to be one of the most promising targets for the development of antitrypanosomal agents.^20−22^ Rhodesain is a cathepsin L-like peptidase, also known as *Tbr*CatL,^23^ which plays essential roles in the disease progression. This protozoal cathepsin is involved in BBB disruption, which promotes the progression of the disease to the neurological stage, and in the immunoevasion process, resulting in an ineffective host immune response.^24,25^ The proteolytic activity mediated by rhodesain occurs through the catalytic triad Cys/His/Asn, which is located in a cleft between the right (R) and left (L) domains,^26^ once the enzyme’s autoinhibitory pro-domain has undergone cleavage.^27^ Considering its key functions, several classes of rhodesain inhibitors have been developed in recent years.^22^ The development of K11777 and K11002 (Figure 1), two potent peptide-based Michael acceptors with which rhodesain was cocrystallized (Protein Data Bank (PDB) ID: 2P7U and 2P86 for K11777 and K11002, respectively),^26,28^ has paved the way toward structure–activity relationship (SAR) studies of irreversible rhodesain inhibitors.^29−36^ At the same time, molecules carrying several electrophilic portions, such as the nitroalkene, nitrile, 3-bromoisoxazoline, (het)arenes, and thiosemicarbazone groups, were developed as reversible rhodesain inhibitors.^37−46^

**Figure 1:**
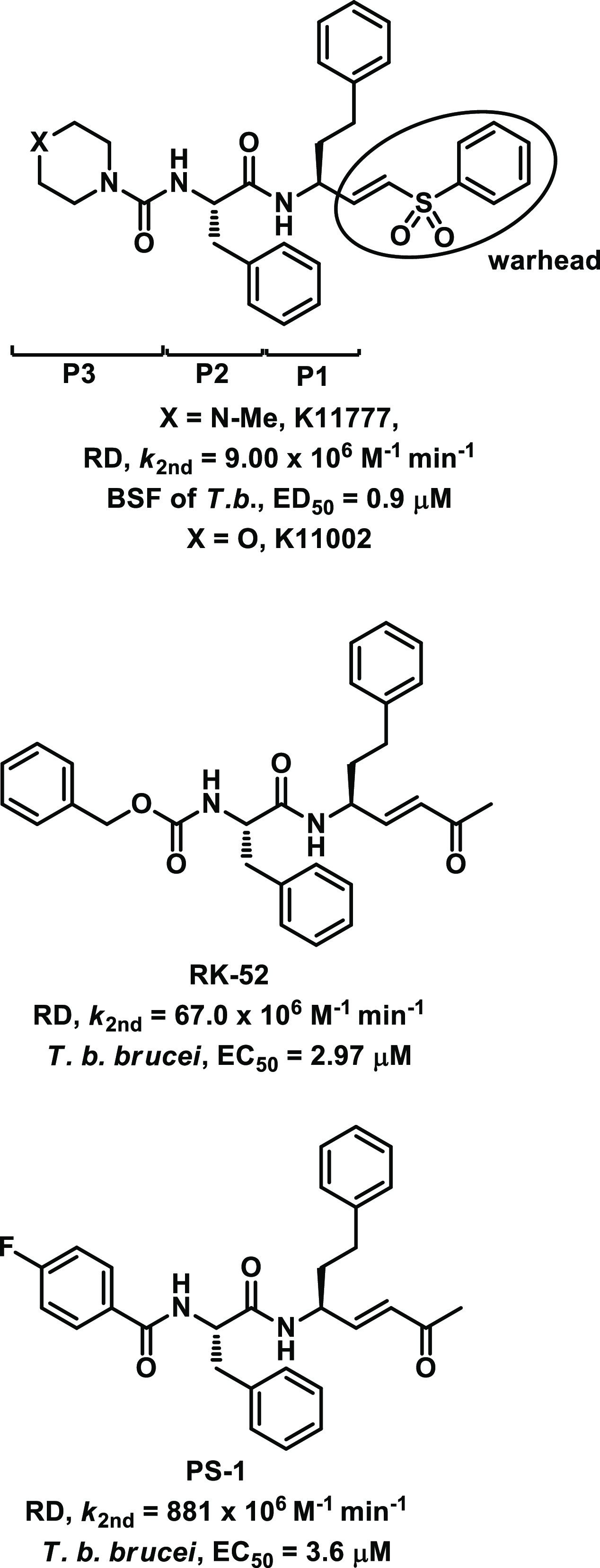
Chemical structures of K11777, K11002, RK-52, and PS-1. Acronyms: RD, rhodesain; BSF, bloodstream form.

In the last decades, we focused our efforts on the development of irreversible rhodesain inhibitors. In particular, the methyl vinyl ketone warhead was identified as the most reactive moiety if compared with vinyl-ester, -sulfone, and -cyano groups.^34,35^ The replacement of the methyl group with bulkier substituents led to analogues with a lower binding affinity toward rhodesain, and for this reason it was unchanged in the subsequent investigations.^31,33^ Similarly to K11777 and K11002, hPhe and Phe at the P1 and P2 positions, respectively, fit well into the respective enzyme pockets, leading to strong binding affinity and potency (Figure 1). At the P3 position, a panel of chemically different substituents was inserted and, with a few exceptions, inhibitors carrying phenyl rings were demonstrated to be very potent rhodesain inhibitors, endowed with single-digit micromolar activity against the protozoa.^31,33^ Differently from the potent vinyl sulfones K11002 and K11777, the concomitant presence of a vinyl ketone warhead and methyl-piperazine and morpholine ring at the P3 position led to poor inhibition.^33^ In light of this, we assumed a strong interdependency between the methyl vinyl ketone warhead and aromatic rings at the P3 position.

Considering the impressive binding activities shown by Michael acceptors reported in Figure 1, in this new set of molecules we decided to maintain the methyl vinyl ketone warhead and the Phe-hPhe lead motif, which are well-fitted in the S2–S1 rhodesain pockets. At the P3 position, a set of variously substituted aromatic rings was inserted through an urea bond (Figure 2). The replacement of the typical peptide bond with the urea function could improve the affinity and the antitrypanosomal activity. In fact, while very potent rhodesain inhibitors have been reported to date, the antitrypanosomal activity was shown to be in the micromolar or sub-micromolar range. Furthermore, the urea bond could be considered as an amide bond bioisostere, possessing more rigidity and stability.^47^ In a SAR study carried out by Patrick et al., the amide-urea bond substitution led to urea derivatives with potent in vitro activity against *T. b. rhodesiense*, high metabolic stability, and moderate brain penetration.^48^ Overall, variously decorated aromatic rings were inserted at the P3 position. In addition to the unsubstituted phenyl ring (**1**, SPR1), we decided to explore the impact of electron-donating groups (EDGs) at the *para* position (i.e., **2** (SPR2) and **3** (SPR3) carrying -OMe and -Me, respectively). The introduction of halogens led to relevant potency enhancement against rhodesain,^33^ and for this reason halogen-containing phenyl rings were inserted (**4** (SPR4), **5** (SPR5), and **6** (SPR6)). To further investigate the role played by halogens in the *para* position, disubstituted 4-chloro-2-methyl- and 4-chloro-2-CF_3_-phenyl rings were introduced (**7** (SPR7) and **8** (SPR8)), because the 4-chloro-2-CF_3_-phenyl ring resulted in being well-tolerated in peptidomimetic rhodesain inhibitors.^42,49^ Lastly, the aromatic region at the P3 position was further expanded through the introduction of a 1-naphthyl ring (**9**, SPR9).

**Figure 2:**
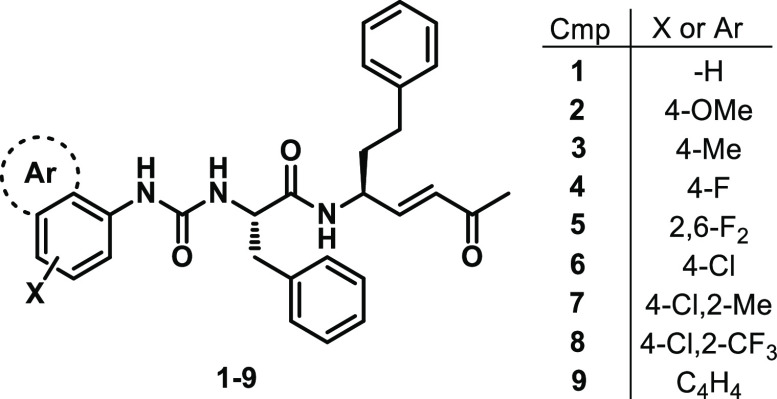
Chemical structure of designed urea derivatives **1**–**9**.

The synthesis of compounds **1**–**9** was carried out in batches following the Boc-chemistry. With the aim to optimize our synthetic pathway previously reported, in which the dipeptidyl vinyl ketones were synthesized starting from the P1 synthon,^50^ followed by the coupling with P2–P3 fragments and warhead incorporation by means of cross-metathesis,^31,33,34^ the new urea derivatives were synthesized following a different approach (Scheme 1). Initially, the commercially available Boc-hPhe-OH **10** was coupled with *N*,*O*-dimethylhydroxylamine hydrochloride **11** in the presence of TBTU and DIPEA, affording the corresponding Weinreb amide **12** in high yields (91%). After that, the peptide backbone P3–P2–P1 was synthesized from the *C*-terminal to N-terminal portion: indeed, the subsequent TFA treatment in DCM led to the deprotected amine TFA salt **13**, which was coupled with Boc-Phe-OH. The obtained dipeptide **14** was newly treated with TFA, and the subsequent reaction with the appropriate differently substituted phenyl isocyanates in alkaline conditions by Et_3_N provided the urea derivatives **16**–**24**. At this point, the reduction of the Weinreb amide by LiAlH_4_ in dry THF led to the corresponding aldehyde analogues, which serve as substrates for the introduction of vinyl methyl ketone warhead by Wittig reaction with 1-(triphenylphosphoranylidene)-2-propanone. All in all, the above-described approach resulted in being widely feasible, and the final products were obtained in a shorter time, with limited use of consumables and slightly better overall yields with respect to the our previously reported synthetic method.^31,33,34^

**Scheme 1:**
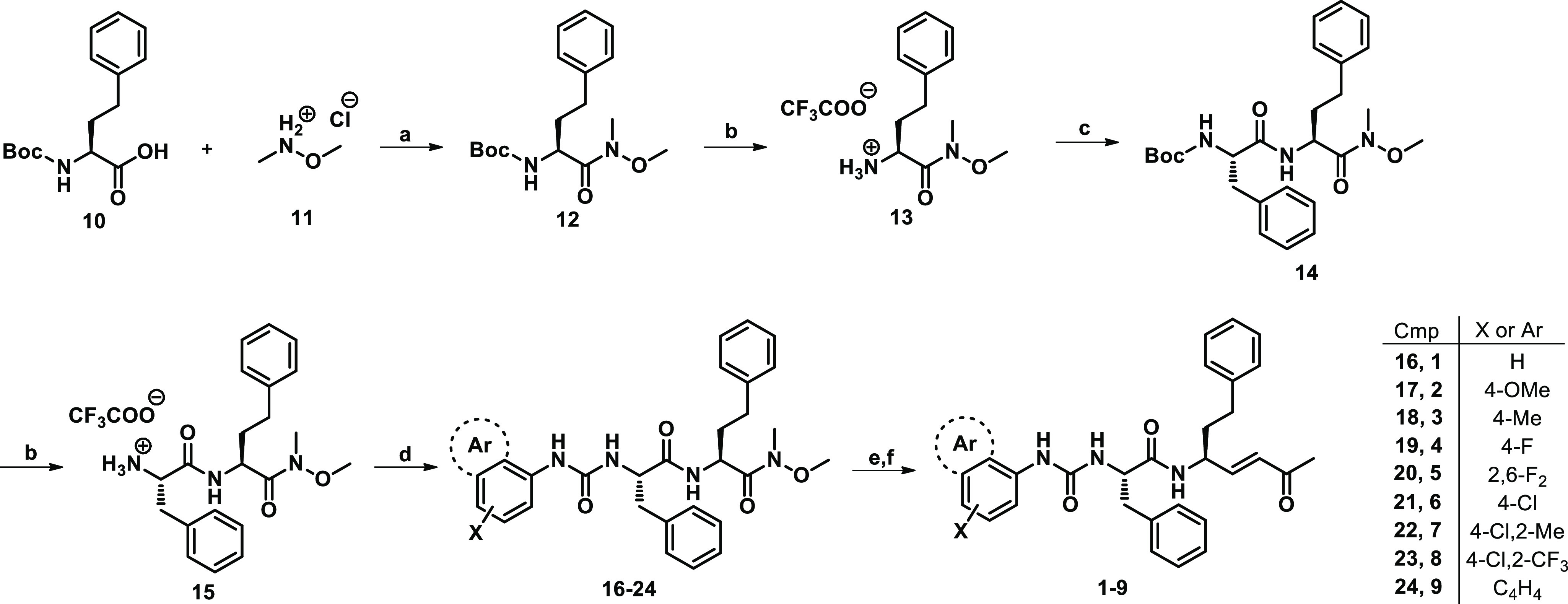
Synthesis of Compounds **1**–**9** Reagents and conditions: (a) TBTU, DIPEA, DCM, 30 min, rt, then **11**, rt, on; (b) TFA/DCM 1:1, rt, TLC monitoring; (c) Boc-Phe-OH, TBTU, DIPEA, DCM, 10 min, rt, then **13**, rt, on; (d) TEA, appropriate Ar-NCO, rt, on; (e) LiAlH_4_, dry THF, 0 °C, TLC monitoring; (f) DCM, Ph_3_PCHCOCH_3_, rt, 2 h.

The biological activity of molecules **1**–**9** against rhodesain was determined by fluorogenic assays in the presence of the appropriate substrate (i.e., Cbz-Phe-Arg-AMC). The irreversible inhibitors could be enzymatically characterized by three kinetic parameters, namely, *k*_inact_, *K*_i_, and *k*_2nd_, which mean the maximum potential rate of covalent bond formation, binding affinity toward the target, and potency of inhibitors, respectively. In more detail, *K*_i_ represents the dissociation constant of the noncovalent enzyme–inhibitor complex [E·I]; meanwhile, *k*_inact_, represents the inactivation rate constant, which defines the covalent enzyme–inhibitor complex (E-I) formation rate. Lastly, the *k*_inact_/*K*_i_ ratio provides the *k*_2nd_ value, which could be considered the best parameter to characterize irreversible inhibitors. Initially, a preliminary screening at 0.1 μM was performed, and DMSO and E-64^51^ were used as the negative and positive control, respectively. Considering the great inhibition shown at the screening concentration (>80%), all of the urea derivatives **1**–**9** were properly diluted and assayed until the minimal percentage inhibition was observed. All of the new Michael acceptors resulted in being very potent rhodesain inhibitors with *K*_i_ values in the nanomolar and sub-nanomolar range (Table 1), and **1**, **3**, and **7** exhibited slightly better *k*_2nd_ values than that of K11777 reported in the literature.^28^ The unsubstituted analogue **1** showed the best binding affinity and potency toward the target (*K*_i_ = 0.15 nM and *k*_2nd_ = 16700 × 10^3^ M^–1^ min^–1^, respectively). Generally, the presence of substituents on the phenyl ring at the P3 position resulted in a slight decrease in binding affinity. The effect of the methyl group in **3** led to *K*_i_ and *k*_2nd_ values that were 2-fold better with respect to the corresponding OMe-containing analogue **2**, whereas **4**, **5**, and **6**, which bear at least a halogen atom, showed similar enzyme inhibitory properties. With regard to the disubstituted analogues, while **7** exhibited a *k*_2nd_ value comparable to that of **1**, the replacement of CH_3_ with CF_3_ in **8** was poorly tolerated. Lastly, the extension of the aromatic region with the introduction of the 1-naphthyl ring in **9** was unproductive in terms of affinity and potency.

**Table 1:**
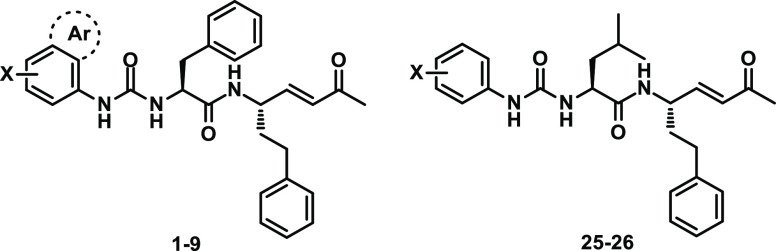
Biological Activity of **1**–**9**, **25**, and **26** against Rhodesain and *T. b. brucei*

		Rhodesain			*T. b. brucei*
compd	X or Ar	*k*_inact_ (min^–1^)	*K*_**i**_ (nM)	*k*_2nd_ (×10^3^ M^–1^ min^–1^)	EC_50_ (μM, 24 h)
**1**	H	0.0021 ± 0.0001	0.15 ± 0.06	16 700 ± 6100	19.79 ± 2.67
**2**	4-OMe	0.0059 ± 0.0014	1.05 ± 0.33	5820 ± 490	2.08 ± 0.22
**3**	4-Me	0.0023 ± 0.0001	0.51 ± 0.38	10 400 ± 7800	1.39 ± 0.23
**4**	4-F	0.0021 ± 0.0002	0.85 ± 0.07	2480 ± 50	1.51 ± 0.08
**5**	2,6-F_2_	0.0022 ± 0.0003	1.13 ± 0.26	2030 ± 230	3.20 ± 0.28
**6**	4-Cl	0.0023 ± 0.0002	0.78 ± 0.13	2990 ± 300	1.50 ± 0.24
**7**	4-Cl,2-Me	0.0053 ± 0.0019	0.51 ± 0.36	14 700 ± 6400	1.65 ± 0.07
**8**	4-Cl,2-CF_3_	0.0060 ± 0.0011	1.04 ± 0.31	6050 ± 790	1.28 ± 0.04
**9**	C_4_H_4_	0.0084 ± 0.0029	2.51 ± 1.51	4170 ± 1360	2.28 ± 0.27
**25**	4-Me	0.0042 ± 0.0001	0.42 ± 0.08	10 360 ± 1590	1.54 ± 0.09
**26**	4-Cl,2-Me	0.0123 ± 0.0060	0.93 ± 0.52	13 980 ± 1320	1.12 ± 0.05
E64^34^		0.0090 ± 0.0004	35 ± 5	261 ± 27	-
K11777^28^				9000	

All the urea derivatives were tested against cultured *T. b. brucei*, which expresses rhodesain similarly to *T. b. rhodesiense*. Unexpectedly, the most potent rhodesain inhibitor **1** showed an EC_50_ value of 19.79 μM (Table 1), whereas the remaining tested compounds exhibited antitrypanosomal activity in the low micromolar range (EC_50_ values ranging from 1.27 to 3.19 μM). Considering the fairly flat SAR concerning the target inhibition, especially in terms of *K*_i_ values, comparable EC_50_ values against the protozoa were analogously expected. The difference in terms of rhodesain inhibition and antitrypanosomal activity observed for **1** could be due to its poor cell membrane permeability. In fact, we assume that the presence of substituents on the phenyl ring at the P3 position, as well as the extension of the aromatic region, could influence the crossing of cell membranes.

In order to validate the role and the importance played by the urea bond and substituents at the P3 position, the Phe at the P2 site was replaced with Leu, which is highly preferred by rhodesain in this position. Considering the *k*_2nd_ values and the antitrypanosomal activity shown by Phe-containing Michael acceptors **1**–**9**, the 4-Me-phenyl and 4-Cl,2-Me-phenyl rings, incorporated in compounds **3** and **7**, respectively, were inserted at position P3. The two new Michael acceptors **25** (SPR46) and **26** (SPR45) were synthesized with the same procedure described in Scheme 1, using Boc-Leu-OH instead of Boc-Phe-OH (Scheme 2). In the biological evaluation (Table 1), Leu-containing analogues **25** and **26** exhibited inhibitory properties comparable to those shown by Phe derivatives **3** and **7** against both rhodesain and protozoa. The introduction of Leu at the P2 site kept the activity toward the enzyme target unchanged and did not influence the cell membrane permeability. With the exception of compound **1**, the ureido derivatives herein reported showed EC_50_ values against the protozoa in the same order of magnitude of lead compounds RK-52 and PS-1,^33,34^ despite a lower inhibition toward rhodesain. This well-known discrepancy in the drug discovery process is generally ascribed to the cell permeability properties.

**Scheme 2:**
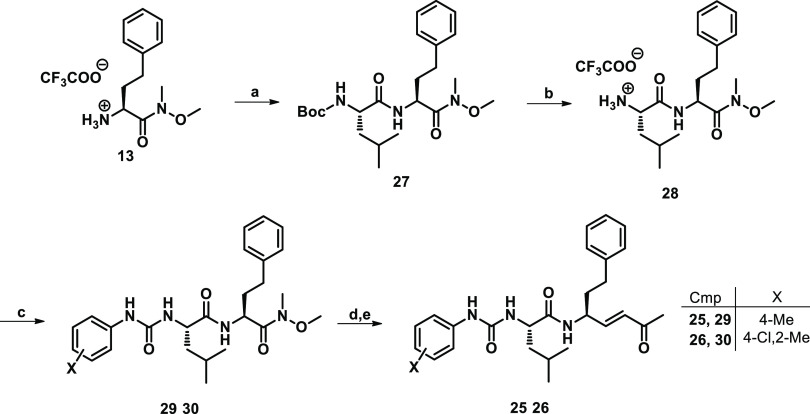
Synthesis of Compounds **25** and **26** Reagents and conditions: (a) Boc-Leu-OH, TBTU, DIPEA, DCM, 30 min, rt, then **13**, rt, on; (b) TFA/DCM 1:1, rt, TLC monitoring; (c) TEA, appropriate Ar-NCO, rt, on; (d) LiAlH_4_, dry THF, 0 °C, TLC monitoring; (e) DCM, Ph_3_PCHCOCH_3_, rt, 2 h.

Considering that some of our previously reported rhodesain inhibitors also showed micromolar activity against the main protease (M^pro^) of SARS-CoV-2,^52^ the novel Michael acceptors were assayed toward the viral protease. However, in the preliminary screening carried out at 100 μM, **1**–**9** exhibited a limited percentage of inhibition, ranging from 33% to 66%. In light of this, dilution assays characterizing the mode of inhibition were not performed. Since poor inhibition was shown by the Phe-containing analogues, the Leu derivatives **25** and **26** were not tested against SARS-CoV-2 M^pro^.

All of the new urea derivatives were tested against human cathepsin L (hCatL), which shares a high percentage of sequence identity with rhodesain (Table S1). With a few exceptions, the new rhodesain inhibitors showed a marginal selectivity toward rhodesain. In particular, while comparable *K*_i_ values between rhodesain and hCatL were observed, in the latter the *k*_inact_ value was 1 order of magnitude less than those displayed in the assays against rhodesain. Overall, the *k*_2nd_ values observed for hCatL inhibition were slightly lower with respect to those against rhodesain. Even if the *k*_2nd_ values toward rhodesain and hCatL differ by only 1 order of magnitude, the new urea derivatives could be well tolerated in animals, due to high levels of mammalian cysteine protease and relative gene expression.^53,54^

To gain major insight into the reasons for the rhodesain inhibitory activity of the new urea derivatives, in silico molecular docking calculations were performed on **1**–**9**, **25**, and **26**, and the obtained data of the most active compound **7** has been reported. This compound was subjected to molecular modeling studies as it features the best compromise between in vitro and in cellulo activity among the newly described compounds. The ligand was covalently docked into rhodesain’s active site using the C25 residue as an anchoring point and employing the covalent docking protocol in AutoDock4 (AD4) software (see the Supporting Information). The same technique was effectively used in other rhodesain inhibitors carrying similar Michael acceptor warheads that form the covalent bond with C25 in the rhodesain.^31,33,34^

Indeed, the predicted binding pose for **7** was similar to the one adopted by the cocrystal K11002 ligand having the P1, P2, and P3 regions gorged at their respective enzyme clefts S1, S2, and S3 (Figure 3A) and by the other structural congeners described by us.^31,33,34^ Notably, the presence of π–π and lipophilic interactions in the S3 pocket (Figure 3B) engaged by the 4-chloro-2-methyl-phenyl group could explain its higher inhibitory potency if compared to K11002 that features a morpholine ring in the same position. In the predicted binding pose, the methyl vinyl ketone warhead is lodged in the polar S1’ subpocket, making positive contacts with H162 (Figure 3B). Here, the backbone NH of the P1 (hPhe) is involved in a H-bond interaction with the backbone CO of D161, and the phenyl ring of the P1(hPhe) is solvent-exposed. The backbone CO of the P2 residue (Phe) is accepting a H-bond from the backbone NH of the W26 residue, while its side chain is lodged inside the hydrophobic S2 cleft that includes various hydrophobic residues such as M68, A138, A208, and L160. The urea linker forms an additional H-bond with the G66 backbone NH and allows projection of the P3 4-chloro-2-methyl-phenyl group into the S3 cleft. As already mentioned, this latter group can establish π–π interaction with F61. This contact should be reinforced by the electron-withdrawing nature of the 4-chloro group, although this effect seems to be counterbalanced by the unfavorable electrostatic interactions established by the same group with the F61 π-electron cloud. Interestingly, this should explain why compounds featuring electron-donating groups (**2** and **3**, see Figure S13) and more negatively charged substituents (**4**, Figure S13) are less potent rhodesain inhibitors. On the contrary, the 2-methyl seems to engage a positive van der Waals interaction with the L67 residue, thereby explaining why compounds featuring more polar groups such as **5** and **8** (see Figure S13) are less potent than **7**. Moreover, the same methyl group seems to hamper the planar conformation of the phenylurea moiety, thereby allowing a proper fitting of the same group into the S3 cleft. All in all, while the 2-methyl substituent in **7** seems to clearly enhance the interactions with the rhodesain S3 pocket, the 4-chloro group should have a mixed effect on the ligand binding.

**Figure 3:**
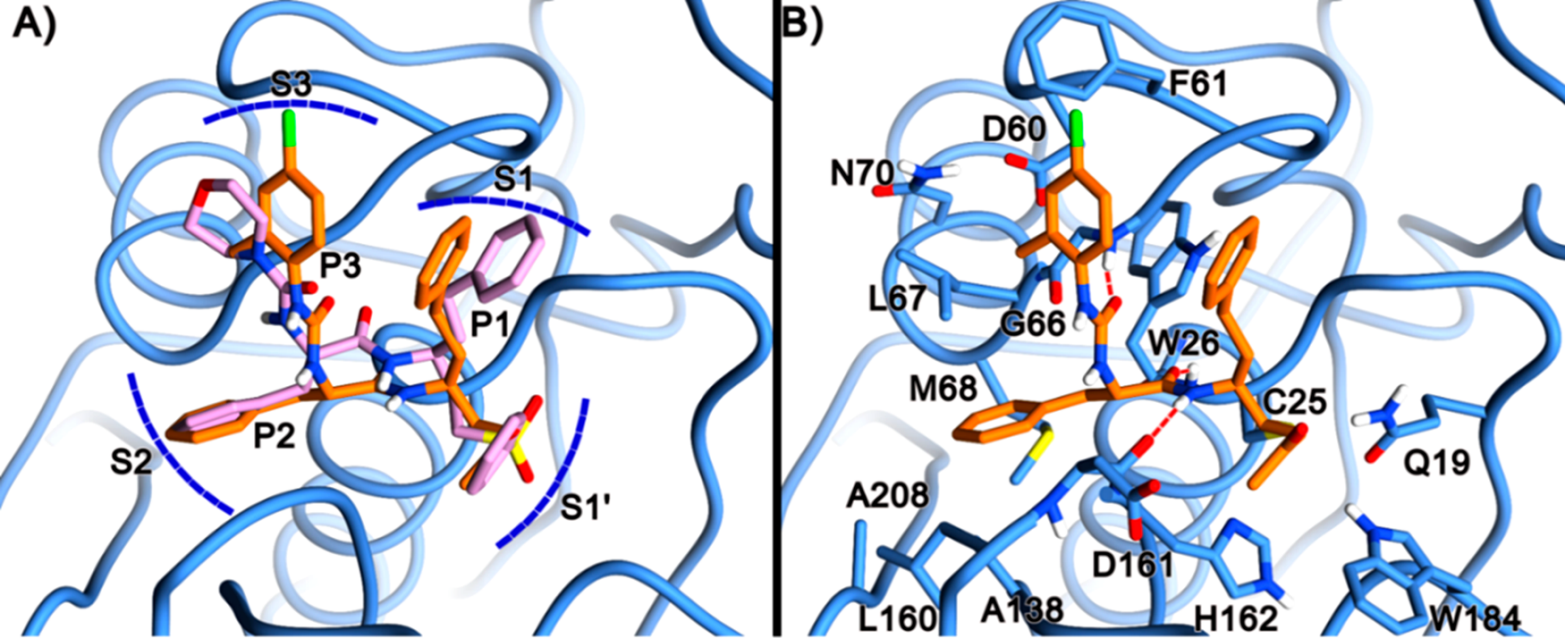
(A) Superimposition of the docked conformations of K11002 (pink) and **7** (orange) in the rhodesain binding site (blue). (B) Predicted theoretical binding pose of **7**/rhodesain and their interactions. The enzyme is depicted in blue ribbons and sticks, and the ligand in orange sticks. Important residues are labeled. H-bonds are shown as red dashed lines. The images were rendered using UCSF Chimera.^55^

This would explain why **1** (see Figure S11), which features an unsubstituted phenyl ring at the P3 site, is the most proficient rhodesain inhibitor of the newly presented series. To gain further insights into the stability of the predicted binding pose and of the described molecular interactions, the **7**/rhodesain complex resulting from the docking experiment was subjected to 100 ns of a molecular dynamics (MD) simulation using Desmond. In this inspection, the ligand of **7** (L-RMSD and L-RMSF) was inspected. If compared to the docking results, the majority of the predicted ligand/protein interactions were preserved during the course of MD (see Figure S14) as **7** displayed fairly good low RMSD fluctuations (see Figure S15). The average RMSD value is 1.36 Å with a standard deviation of 0.34, whereas the average RMSF broken down atom-by-atom value is 0.89 Å with a standard deviation of 0.53, as shown in the Supporting Information (see Figure S16).

In this SAR study, a small panel of Michael acceptors was developed as potential rhodesain inhibitors and antitrypanosomal agents. The novel analogues **1**–**9**, **25**, and **26** carry a peptide backbone Phe/Leu-hPhe and the methyl vinyl ketone warhead, which represent the lead motif and reactive electrophilic portion, respectively. At the P3 position, differently decorated aromatic rings were anchored to the lead motif through a urea bond. All the urea derivatives exhibited potent inhibitory activity toward rhodesain, with *K*_i_ values in the nanomolar and sub-nanomolar ranges, and **1**, **3**, **7**, **25**, and **26** showed comparable potency to that of K11777. The substituent-containing analogues on the phenyl ring at the P3 position displayed single-digit EC_50_ values against the protozoa. No significant differences were observed when Leu was incorporated at the P2 site instead of Phe. The best compromise in terms of activity against both the enzyme and protozoa was observed in **7**, which showed potent rhodesain inhibition and EC_50_ values in the low micromolar range. In the future, compound **7** could represent an interesting lead compound for further investigation of the S3 rhodesain pocket and the development of rhodesain inhibitors and anti-HAT agents.

## Supporting Information Available

Detailed description of the synthesis of the intermediates and final products, and relative characterization; description of the biological evaluation toward rhodesain, SARS-CoV-2 M^pro^, hCatL and protozoan; detailed description of the molecular modeling procedures docking results, molecular dynamics analysis; NMR spectra of the final compounds. hCatL biological activity (PDF)

## Abbreviations

HAT: Human African Trypanosomiasis
NTS: Neglected Tropical Disease
BBB: blood-brain barrier
CNS: central nervous system
FDA: U.S. Food and Drug Administration
WHO: World Health Organization
DNDi: Drugs for Neglected Diseases Initiative
NECT: Nifurtimox-Eflornithine Combination Therapy
PDB: Protein Data Bank
SAR: structure–activity relationship
EDG: electron-donating group
AMC: 7-Amino-4-methylcoumarin
hCatL: human Cathepsin L.
