# Supplementary material for: Development of Urea-Bond-Containing Michael Acceptors as Antitrypanosomal Agents Targeting Rhodesain
Source: ACS Med Chem Lett. 2022 Jun 30;13(7):1083–90. doi: 10.1021/acsmedchemlett.2c00084 (PMC9290002; doi:10.1021/acsmedchemlett.2c00084)
Supplement: Supplementary file 1 — ml2c00084_si_001.pdf [file ml2c00084_si_001.pdf]

Supporting Information for:

Development of Urea Bond-containing Michael Acceptors as Antitrypanosomal Agents Targeting Rhodesain

Santo Previti,[1,*] Roberta Ettari,[1] Elsa Calcaterra,[1] Carla Di Chio,[1] Rahul Ravichandran,[2] Collin Zimmer,[3] Stefan Hammerschmidt,[3] Annika Wagner,[4] Marta Bogacz,[4] Sandro Cosconati,[2] Tanja Schirmeister,[3] Maria Zappalà[1]

[1]*Department of Chemical, Biological, Pharmaceutical and Environmental Sciences, University of Messina, Viale Ferdinando Stagno d'Alcontres 31, 98166 Messina, Italy.*

[2]*DiSTABiF, University of Campania "Luigi Vanvitelli", Via Vivaldi 43, 81100 Caserta, Italy.*

[3]*Institute of Pharmaceutical and Biomedical Sciences, University of Mainz, Staudingerweg 5, 55128 Mainz, Germany.*

[4]*Institute of Organic Chemistry & Macromolecular Chemistry, Friedrich-Schiller-University of Jena, Humboldtstraße 10, 07743 Jena, Germany.*

AUTHOR INFORMATION

Corresponding Author: Santo Previti - Department of Chemical, Biological, Pharmaceutical and Environmental Sciences, University of Messina, Italy. ORCID: 0000-0001-8473-3321. Phone: +39 090 676 6411. E-mail: spreviti@unime.it

SUPPLEMENTAL TABLE OF CONTENT

Experimental……S5

Chemistry……S5

(*S*)-tert-Butyl (1-(methoxy(methyl)amino)-1-oxo-4-phenylbutan-2-yl)carbamate (**12**)……S5

(*S*)-1-(methoxy(methyl)amino)-1-oxo-4-phenylbutan-2-aminium 2,2,2-trifluoroacetate (**13**)……S5

tert-Butyl ((*S*)-1-(((*S*)-1-(methoxy(methyl)amino)-1-oxo-4-phenylbutan-2-yl)amino)-1-oxo-3-phenylpropan-2-yl)carbamate (**14**)……S6

tert-Butyl ((*S*)-1-(((*S*)-1-(methoxy(methyl)amino)-1-oxo-4-phenylbutan-2-yl)amino)-4-methyl-1-oxopentan-2-yl)carbamate (**27**)……S6

(*S*)-1-(((*S*)-1-(Methoxy(methyl)amino)-1-oxo-4-phenylbutan-2-yl)amino)-1-oxo-3-phenylpropan-2-aminium 2,2,2-trifluoroacetate (**15**)……S6

(*S*)-1-(((*S*)-1-(Methoxy(methyl)amino)-1-oxo-4-phenylbutan-2-yl)amino)-4-methyl-1-oxopentan-2-aminium 2,2,2-trifluoroacetate (**28**)……S6

General procedure for the synthesis of urea intermediates **16-24, 29,** and **30**……S7

S)-N-Methoxy-*N*-methyl-4-phenyl-2-((*S*)-3-phenyl-2-(3-phenylureido)propanamido) butanamide (**16**)……S7

(*S*)-*N*-Methoxy-2-((*S*)-2-(3-(4-methoxyphenyl)ureido)-3-phenylpropanamido)-N-methyl-4-phenylbutanamide (**17**)……S7

(*S*)-*N*-Methoxy-*N*-methyl-4-phenyl-2-((*S*)-3-phenyl-2-(3-(*p*-tolyl)ureido)propanamido) butanamide (**18**)……S7

(*S*)-2-((*S*)-2-(3-(4-Fluorophenyl)ureido)-3-phenylpropanamido)-*N*-methoxy-*N*-methyl-4-phenylbutanamide (**19**)……S8

(*S*)-2-((*S*)-2-(3-(2,6-Difluorophenyl)ureido)-3-phenylpropanamido)-*N*-methoxy-*N*-methyl-4-phenylbutanamide (**20**)……S8

(*S*)-2-((*S*)-2-(3-(4-Chlorophenyl)ureido)-3-phenylpropanamido)-*N*-methoxy-*N*-methyl-4-phenylbutanamide (**21**)……S8

(*S*)-2-((*S*)-2-(3-(4-Chloro-2-methylphenyl)ureido)-3-phenylpropanamido)-*N*-methoxy-*N*-methyl-4-phenylbutanamide (**22**)……S8

(*S*)-2-((*S*)-2-(3-(4-Chloro-2-(trifluoromethyl)phenyl)ureido)-3-phenylpropanamido)-*N*-methoxy-*N*-methyl-4-phenylbutanamide (**23**)……S9

(*S*)-*N*-Methoxy-*N*-methyl-2-((*S*)-2-(3-(naphthalen-1-yl)ureido)-3-phenylpropanamido)-4-phenylbutanamide (**24**)……S9

(*S*)-*N*-((*S*)-1-(Methoxy(methyl)amino)-1-oxo-4-phenylbutan-2-yl)-4-methyl-2-(3-(*p*-tolyl)ureido)pentanamide (**29**)……S9

(*S*)-2-(3-(4-Chloro-2-methylphenyl)ureido)-*N*-((*S*)-1-(methoxy(methyl)amino)-1-oxo-4-phenylbutan-2-yl)-4-methylpentanamide (**30**)……S9

General procedure for the synthesis of final compounds **1-9**, **25**, and **26**……S10

(*S*)-*N*-((*S*,*E*)-6-Oxo-1-phenylhept-4-en-3-yl)-3-phenyl-2-(3-phenylureido)propenamide (**1**)……...S10
(*S*)-2-(3-(4-Methoxyphenyl)ureido)-*N*-((*S*,*E*)-6-oxo-1-phenylhept-4-en-3-yl)-3-phenylpropanamide (**2**) ……………………………………………………………………….S10
(*S*)-*N*-((*S*,*E*)-6-Oxo-1-phenylhept-4-en-3-yl)-3-phenyl-2-(3-(*p*-tolyl)ureido)propanamide (**3**)……S10
(*S*)-2-(3-(4-Fluorophenyl)ureido)-*N*-((*S*,*E*)-6-oxo-1-phenylhept-4-en-3-yl)-3-phenylpropanamide (**4**)……………………………………………………………………….S11
(*S*)-2-(3-(2,6-Difluorophenyl)ureido)-*N*-((*S*,*E*)-6-oxo-1-phenylhept-4-en-3-yl)-3-phenylpropanamide (**5**) S11
(*S*)-2-(3-(4-Chlorophenyl)ureido)-*N*-((*S*,*E*)-6-oxo-1-phenylhept-4-en-3-yl)-3-Phenylpropanamide (**6**)...S11
(*S*)-2-(3-(4-Chloro-2-methylphenyl)ureido)-*N*-((*S*,*E*)-6-oxo-1-phenylhept-4-en-3-yl)-3-phenylpropanamide (**7**) S12
(*S*)-2-(3-(4-Chloro-2-(trifluoromethyl)phenyl)ureido)-*N*-((*S*,*E*)-6-oxo-1-phenylhept-4-en-3-yl)-3-phenylpropanamide (**8**) S12
(*S*)-2-(3-(Naphthalen-1-yl)ureido)-*N*-((*S*,*E*)-6-oxo-1-phenylhept-4-en-3-yl)-3-phenylpropanamide (**9**) S12
(*S*)-4-Methyl-*N*-((*S*,*E*)-6-oxo-1-phenylhept-4-en-3-yl)-2-(3-(p-tolyl)ureido)pentanamide (25).......S12
(*S*)-2-(3-(4-chloro-2-methylphenyl)ureido)-4-methyl-*N*-((*S*,*E*)-6-oxo-1-phenylhept-4-en-3-yl)pentanamide (26)...S13
Biological evaluation……………………………………………………………………………..S14
Enzyme assays towards rhodesain……………………………………………………………….S14
Chart S11. Equations 1, 2, and 3…………………………………………………………………S14
Enzyme assays towards hCatL…..………………………………………………………………S14
Enzyme assays towards SARS-CoV-2 M^{pro}……………………………………………………..S14
Antitrypanosomal activity………………………………………………………………………..S14
Molecular Modeling………………………………………………………………………………S16
Docking……………………………………………………………………………………………..S16
Molecular Dynamics………………………………………………………………………………..S16
References………………………………………………………………………………………...S18
NMR Spectra……………………………………………………………………………………..S20
Figure S1. ^{1}H NMR spectrum of compound **1**…………………………………………………...S20
Figure S2. COSY spectrum of compound **1**…………………………………………………….S20
Figure S3. ^{1}H NMR spectrum of compound **2**…………………………………………………...S21
Figure S4. ^{1}H NMR spectrum of compound **3**…………………………………………………...S21
Figure S5. ^{1}H NMR spectrum of compound **4**…………………………………………………...S22
Figure S6. ^{1}H NMR spectrum of compound **5**…………………………………………………...S22
Figure S7. ^{1}H NMR spectrum of compound **6**…………………………………………………...S23

Figure S8. ^{1}H NMR spectrum of compound **7**……………………………………………………...S23
Figure S9. ^{1}H NMR spectrum of compound **8**……………………………………………………...S24
Figure S10. ^{1}H NMR spectrum of compound **9**…………………………………………………….S24
Figure S11. ^{1}H NMR of compound **25**……………………………………………………………..S25
Figure S12. ^{1}H NMR of compound **26**……………………………………………………………..S25
Figure S13. docking results for compounds **1-8**, **9**, **25**, and **26**…………………………………….S26
Figure S14. MD interaction diagrams……………………..……………………………………..S27
Figure S15. RMSD (Å) plot over time..……………………………………………………….S27
Figure S16. L-RMSF ……………………..…………………………………………………….S28
Table S1. Biological evaluation towards hCatL……………………………………………………S29

EXPERIMENTAL SECTION

Chemistry.

All reagents and solvents were purchased from commercial suppliers. Boc-hPhe-OH, *N*,*O*-dimethylhydroxylamine, TBTU and Boc-Phe-OH were obtained from Fluorochem. The aryl isocyanates, TEA, TFA, $LiAlH_4$, and 1-(triphenylphosphoranylidene)-2-propanone were obtained from Merck, as well as silica gel 60 F254 plates and silica gel (200-400 mesh) employed for TLC and column chromatography, respectively. 2,4-Dinitrophenylhydrazine TLC stain was used to detect the aldehyde intermediates. In all the manipulations, no unexpected or unusually high safety hazards were encountered. All the ^{1}H and ^{13}C spectra were performed on a Varian 500 MHz provided with a ONE NME probe operating at 499.74 and 125.73 MHz for ^{1}H and ^{13}C, respectively. Deuterated solvents (i.e., $CDCl_3$, MeOD, and DMSO-d6) were obtained from Merck and the signal of the solvents was used as the internal standard. Splitting patterns are described as singlet (s), doublet (d), doublet of doublet (dd), triplet (t), multiplet (m) and broad singlet (bs). Chemical shifts were expressed in ppm and coupling constants (J) in Hz. Elemental analyses were performed on a C. Erba model 1106 (elemental analyzer for C, H and N) apparatus, and the found results are within ±0.4% of the theoretical values.

***(S)*-tert-Butyl (1-(methoxy(methyl)amino)-1-oxo-4-phenylbutan-2-yl)carbamate (12)** In a round bottom flask, Boc-hPhe-OH **10** (1 eq.) was solubilized in DCM (10 mL/mmol). Subsequently, the flask was cooled down with an ice bath at 0°C and TBTU (1.1 eq.) and DIPEA (3 eq.) were added. The reaction was maintained in stirring for 30 min. After this time, *N*,*O*-dimethylhydroxylamine hydrochloride **11** (1.2 eq.) was added portion-wise, pH monitored (>7), the ice-bath removed, and the reaction was stirred on at rt. DCM was removed and the resulting residue was diluted in EtOAc and washed with 1 M HCl (x 3), $NaHCO_3$ saturated solution (x 3), and brine (x 3), dried over Na_2SO_4, filtered and concentrated. The desired compound was purified by column chromatography using light petroleum/EtOAc (4:1) as eluent. Yield = 91%; R_f = 0.47 (light petroleum/EtOAc, 4:1); Consistency = colorless oily liquid. ^{1}H NMR (500 MHz, $CDCl_3$): δ = 1.45 (s, 9H), 1.79-1.88 (m, 1H), 1.98-2.07 (m, 1H), 2.62-2.70 (m, 1H), 2.71-2.78 (m, 1H), 3.17 (s, 3H), 3.63 (s, 3H), 4.63-4.75 (m, 1H), 5.22 (d, J = 8.4 Hz, 1H), 7.16-7.22 (m, 3H), 7.25-7.30 (m, 2H). NMR data are in agreement to those reported in the literature.[1]

(*S*)-1-(methoxy(methyl)amino)-1-oxo-4-phenylbutan-2-aminium 2,2,2-trifluoroacetate (13) In a round bottom flask, compound **12** (565 mg, 1.75 mmol) was solubilized in DCM (1.8 mL) at rt. Subsequently, TES (90 µL) and TFA (1.8 mL, drop-wise) were added. The reaction was kept in stirring for 1h. After this time, the TLC (light petroleum/ EtOAc, 3:2) showed the absence of starting material. DCM was easily evaporated, while TFA was co-evaporated with toluene (x 3), $CHCl_3$ (x 1) and Et_2O (x 1). The resulting residue was triturated in Et_2O and used for the next step without purification. Yield = 97%; Consistency = white powder. ^{1}H NMR (500 MHz, MeOD): δ = 2.08-2.24 (m, 2H), 2.67-2.83 (m, 2H), 3.21 (s, 3H), 3.64 (s, 3H), 4.26-4.31 (m, 1H), 7.18-7.31 (m, 3H), 7.28-7.33 (m, 2H).

tert-Butyl ((*S*)-1-(((*S*)-1-(methoxy(methyl)amino)-1-oxo-4-phenylbutan-2-yl)amino)-1-oxo-3-phenylpropan-2-yl)carbamate (14) In a round bottom flask (A), Boc-Phe-OH (1.5 eq.) was solubilized in DCM (5 mL/mmol) and TBTU (1.5 eq.) and DIPEA (1 eq.) were added. The flask was cooled down at 0°C and kept in stirring. In a second flask (B) containing compound **13** (1 eq.), DCM (5 mL/mmol) and DIPEA (3 eq.) were added at rt. The resulting solution was stirred for a few minutes and subsequently added drop-wise to the flask A. pH was checked (>7), the ice-bath removed, and the reaction was stirred on at rt. DCM was removed and the resulting residue was diluted in EtOAc and washed with 1 M HCl (x 3), $NaHCO_3$ saturated solution (x 3), and brine (x 3), dried over Na_2SO_4, filtered and concentrated. The desired compound was purified by column chromatography using light petroleum/EtOAc (3:2) as eluent. Yield = 90%; R_f = 0.29 (light petroleum/EtOAc, 3:2); Consistency = white powder. ^{1}H NMR (500 MHz, $CDCl_3$): δ = 1.41 (s, 9H), 1.82-1.93 (m, 1H), 2.00-2.10 (m, 1H), 2.53-2.67 (m, 2H), 2.99-3.12 (m, 2H), 3.15 (s, 3H), 3.63 (s, 3H), 4.30-4.42 (m, 1H), 4.86 (d, J = 6.1 Hz, 1H), 4.92-5.02 (m, 1H), 6.57 (d, J = 7.6 Hz, 1H), 7.15-7.31 (m, 10H).

tert-Butyl ((S)-1-(((S)-1-(methoxy(methyl)amino)-1-oxo-4-phenylbutan-2-yl)amino)-4-methyl-1-oxopentan-2-yl)carbamate (27) Compound **27** was synthesized following the same procedure described for intermediate **14**, and Boc-Leu-OH was used instead of Boc-Phe-OH. The desired compound was purified by column chromatography using light petroleum/EtOAc (7:3) as eluent. Yield = 76%; R_f = 0.26 (light petroleum/EtOAc, 7:3); Consistency = white powder. ^{1}H NMR (500 MHz, $CDCl_3$): δ = 0.93 (d, J = 4.7 Hz, 3H), 0.94 (d, J = 4.9 Hz, 3H), 1.44 (s, 9H), 1.46-1.50 (m, 1H), 1.58-1.73 (m, 2H), 1.87-1.97 (m, 1H), 2.02-2.13 (m, 1H), 2.58-2.75 (m, 2H), 3.16 (s, 3H), 3.62 (s, 3H), 4.09-4.19 (m, 1H), 4.88 (d, J = 8.2 Hz, 1H), 4.92-5.03 (m, 1H), 6.75 (d, J = 8.4 Hz, 1H), 7.15-7.22 (m, 3H), 7.24-7.30 (m, 2H).

(*S*)-1-(((*S*)-1-(Methoxy(methyl)amino)-1-oxo-4-phenylbutan-2-yl)amino)-1-oxo-3-phenylpropan-2-aminium 2,2,2-trifluoroacetate (15) Compound **15** was synthesized following the same procedure employed for compound **13** starting from intermediate **14**. TLC monitoring was carried out using light petroleum/EtOAc (1:1) as eluent. Yield = 98%; Consistency = pale yellow powder; ^{1}H NMR (500 MHz, MeOD): δ = 1.88-1.97 (m, 1H), 2.03-2.13 (m, 1H), 2.57-2.77 (m, 2H), 2.73 (dd, J = 13.7, 9.3 Hz, 1H), 3.17 (s, 3H), 3.26 (dd, J = 13.7, 3.6 Hz, 1H), 3.61 (dd, J = 9.2, 3.6 Hz, 1H), 3.68 (s, 3H), 4.99-5.09 (m, 1H), 7.15-7.35 (m, 10H), 7.88 (d, J = 8.7 Hz, 1H); ^{13}C NMR (126 MHz, MeOD): δ = 31.71, 32.02, 34.07, 40.97, 48.46, 56.52, 61.50, 125.98, 126.81, 128.36, 128.49, 128.69, 129.31, 137.80, 141.10, 172.41, 174.20.

(*S*)-1-(((*S*)-1-(Methoxy(methyl)amino)-1-oxo-4-phenylbutan-2-yl)amino)-4-methyl-1-oxopentan-2-aminium 2,2,2-trifluoroacetate (28) Compound **28** was synthesized following the same procedure employed for compound **13** starting from intermediate **27**. TLC monitoring was carried out using light petroleum/EtOAc (1:1) as eluent. Yield = 97%; Consistency = pale yellow powder; ^{1}H NMR (500 MHz, MeOD): δ = 1.01 (d, J = 3.4 Hz, 3H), 1.03 (d, J = 3.6 Hz, 3H), 1.62-1.71 (m, 1H), 1.72-1.82 (m, 2H), 1.90-2.00 (m, 1H), 2.01-2.12 (m, 1H), 2.58-2.69 (m, 1H), 2.76-2.86 (m, 1H), 3.15 (s, 3H), 3.60 (s, 3H), 3.92-3.96 (m, 1H), 4.80-4.87 (m, 1H), 7.16-7.25 (m, 3H), 7.25-7.31 (m, 2H).

1.1.1 General procedure for the synthesis of urea intermediates 16-24, 29, and 30.

In a round bottom flask, intermediate **15** or **28** (1.2 eq.) was solubilized in dry THF (2 mL/mmol), and TEA (1.3 eq.) was added, pH checked (>7) and the reaction was maintained in stirring for 5 min at rt. After that, the appropriate isocyanate (1 eq.) was diluted in dry THF (same volume employed to solubilize compound **15**/**28**) and added drop-wise to the reaction, which was kept in stirring on at rt. A few drops of 1 M HCl were added to the flask, and the solution was moved in a separatory funnel. A few mL of 1 M HCl were added and the organic phase was extracted with EtOAc (x 3), dried over Na_2SO_4 and concentrated *in vacuo*. The desired compound was purified by column chromatography using the appropriate eluent (see details in the below section).

(*S*)-*N*-Methoxy-*N*-methyl-4-phenyl-2-((*S*)-3-phenyl-2-(3-phenylureido)propanamido)butanamide (16) Eluent mixture = light petroleum/EtOAc (2:3); R_f = 0.38 light petroleum/EtOAc (2:3); Yield = 79%; Consistency = white powder; ^{1}H NMR (500 MHz, $CDCl_3$): δ = 1.79-1.90 (m, 1H), 1.95-2.05 (m, 1H), 2.49-2.57 (m, 1H), 2.58-2.64 (m, 1H), 2.95 (dd, J = 14.0, 8.2 Hz, 1H), 3.11 (s, 3H), 3.12 (dd, J = 13.9, 6.0 Hz, 1H), 3.54 (s, 3H), 4.82 (q, J = 8.0 Hz, 1H), 4.89-4.98 (m, 1H), 6.50 (d, J = 7.9 Hz, 1H), 6.90 (t, J = 7.3 Hz, 1H), 7.00-7.22 (m, 14H), 7.47 (d, J = 6.6 Hz, 1H), 7.70 (s, 1H). ^{13}C NMR (126 MHz, $CDCl_3$): δ = 31.52, 32.17, 33.63, 38.93, 49.35, 55.38, 61.46, 119.89, 122.81, 125.93, 126.77, 128.28, 128.44, 128.83, 129.35, 136.80, 138.85, 140.72, 155.62, 172.03, 173.18.

(*S*)-*N*-Methoxy-2-((*S*)-2-(3-(4-methoxyphenyl)ureido)-3-phenylpropanamido)-*N*-methyl-4-phenylbutanamide (17) Eluent mixture = light petroleum/EtOAc (2:3); R_f = 0.19 light petroleum/EtOAc (2:3); Yield = 79%; Consistency = yellow powder; ^{1}H NMR (500 MHz, $CDCl_3$): δ = 1.77-1.91 (m, 1H), 1.94-2.04 (m, 1H), 2.45-2.68 (m, 2H), 2.58-2.64 (m, 1H), 2.95 (dd, J = 13.8, 8.1 Hz, 1H), 3.11 (dd, J = 13.8, 6.0 Hz, 1H), 3.13 (s, 3H), 3.55 (s, 3H), 3.69 (s, 3H), 4.79 (q, J = 6.0 Hz, 1H), 4.87-4.98 (m, 1H), 6.21 (bs, 1H), 6.63 (d, J = 7.6 Hz, 2H), 7.00 (d, J = 7.5 Hz, 2H), 7.03-7.22 (m, 10H), 7.43 (bs, 1H).^{13}C NMR (126 MHz, $CDCl_3$): δ = 172.95, 172.01, 156.14, 155.95, 140.81, 136.85, 131.45, 129.35, 128.45, 128.43, 128.26, 126.74, 125.90, 122.90, 114.15, 61.46, 55.38, 55.29, 49.20, 38.79, 33.71, 32.12, 31.51.

(*S*)-*N*-Methoxy-*N*-methyl-4-phenyl-2-((*S*)-3-phenyl-2-(3-(*p*-tolyl)ureido)propanamido)butanamide (18) Eluent mixture = light petroleum/EtOAc (1:1); R_f = 0.28 light petroleum/EtOAc (1:1); Yield = 72%; Consistency = white powder; ^{1}H NMR (500 MHz, $CDCl_3$): δ = 1.79-1.88 (m, 1H), 1.94-2.04 (m, 1H), 2.21 (s, 3H), 2.47-2.55 (m, 1H), 2.56-2.64 (m, 1H), 2.94 (dd, J = 13.9, 8.2 Hz, 1H), 3.11 (dd, J = 13.8, 6.0 Hz, 1H), 3.12 (s, 3H), 3.54 (s, 3H), 4.80 (q, J = 6.0 Hz, 1H), 4.88-4.95 (m, 1H), 6.36 (d, J = 7.5 Hz, 1H), 6.88 (d, J = 8.3 Hz, 2H), 7.00 (d, J = 8.3 Hz, 2H), 7.05 (d, J = 7.1 Hz, 2H), 7.07-7.19 (m, 8H), 7.46 (d, J = 7.1 Hz, 1H), 7.57 (s, 1H). ^{13}C NMR (126 MHz, $CDCl_3$): δ = 20.83, 31.62, 32.25, 33.79, 38.98, 49.37, 55.43, 61.58, 120.60, 126.01, 126.85, 128.37, 128.54, 128.57, 129.49, 129.46, 132.61, 136.18, 136.97, 140.91, 155.93, 172.12, 173.16.

(*S*)-2-((*S*)-2-(3-(4-Fluorophenyl)ureido)-3-phenylpropanamido)-*N*-methoxy-*N*-methyl-4-phenylbutanamide (19) Eluent mixture = light petroleum/EtOAc (1:1); R_f = 0.22 light petroleum/EtOAc (1:1); Yield = 80%; Consistency = white powder; ^{1}H NMR (500 MHz, $CDCl_3$): δ = 1.79-1.89 (m, 1H), 1.96-2.06 (m, 1H), 2.48-2.57 (m, 1H), 2.58-2.65 (m, 1H), 2.93 (dd, J = 13.6, 8.5 Hz, 1H), 3.11 (dd, J = 13.6, 6.0 Hz, 1H), 3.14 (s, 3H), 3.56 (s, 3H), 4.75-4.85 (m, 1H), 4.88-4.97 (m, 1H), 6.43 (bs, 1H), 6.72 (t, J = 8.6 Hz, 2H), 6.98-7.08 (m, 4H), 7.08-7.22 (m, 8H), 7.44 (bs, 1H), 7.62 (d, J = 12.7 Hz, 1H). ^{13}C NMR (126 MHz, $CDCl_3$): δ = 31.53, 32.19, 33.69, 38.92, 49.40, 55.42, 61.46, 115.29 (d, J = 22.4 Hz), 121.64 (d, J = 6.8 Hz), 126.02, 126.86, 128.31, 128.39, 128.48, 129.29, 134.67, 136.68, 140.60, 155.68, 158.66 (d, J = 242.4 Hz), 171.97, 173.28.

(*S*)-2-((*S*)-2-(3-(2,6-Difluorophenyl)ureido)-3-phenylpropanamido)-*N*-methoxy-*N*-methyl-4-phenylbutanamide (20) Eluent mixture = light petroleum/EtOAc (1:1); R_f = 0.41 light petroleum/EtOAc (1:1); Yield = 49%; Consistency = white powder; ^{1}H NMR (500 MHz, $CDCl_3$): δ = 1.75-1.86 (m, 1H), 1.91-2.01 (m, 1H), 2.45-2.53 (m, 1H), 2.54-2.61 (m, 1H), 3.05 (d, J = 6.8 Hz, 2H), 3.10 (s, 3H), 3.52 (s, 3H), 4.73 (q, J = 6.7 Hz, 1H), 4.87-4.95 (m ,1H), 6.42 (d, J = 7.8 Hz, 1H), 6.82 (t, J = 8.0 Hz, 2H), 7.02-7.21 (13H). ^{13}C NMR (126 MHz, $CDCl_3$): δ = 31.55, 32.18, 34.01, 39.06, 49.28, 55.64, 61.54, 111.67 (dd, J = 19.0, 4.8 Hz), 115.36 (t, J = 16.1 Hz), 126.01, 126.48 (t, J = 10.0 Hz), 126.86, 128.36, 128.53, 128.56, 129.57, 136.77, 140.98, 155.10, 158.14 (dd, J = 249.7, 4.9 Hz), 171.92, 172.23.

(*S*)-2-((*S*)-2-(3-(4-Chlorophenyl)ureido)-3-phenylpropanamido)-*N*-methoxy-*N*-methyl-4-phenylbutanamide (21) Eluent mixture = light petroleum/EtOAc (1:1); R_f = 0.23 light petroleum/EtOAc (1:1); Yield = 82%; Consistency = white powder; ^{1}H NMR (500 MHz, $CDCl_3$): δ = 1.79-1.89 (m, 1H), 1.96-2.06 (m, 1H), 2.49-2.57 (m, 1H), 2.58-2.67 (m, 1H), 2.90 (dd, J = 14.0, 8.5 Hz, 1H), 3.11 (dd, J = 14.0, 5.5 Hz, 1H), 3.15 (s, 3H), 3.57 (s, 3H), 4.82 (dd, J = 13.8, 8.1 Hz, 1H), 4.88-4.99 (m, 1H), 6.63 (d, J = 7.9 Hz, 1H), 6.93-6.97 (m, 2H), 6.98-7.02 (m, 2H), 7.02-7.06 (m, 2H), 7.08-7.20 (m, 8H), 7.56 (d, J = 6.6 Hz, 1H), 7.76 (bs, 1H). ^{13}C NMR (126 MHz, $CDCl_3$): δ = 31.69, 32.37, 33.75, 39.13, 49.63, 55.57, 61.63, 120.74, 126.21, 127.03, 127.67, 128.47, 128.51, 128.61, 128.78, 129.41, 136.75, 137.57, 140.65, 155.56, 172.14, 173.71.

(*S*)-2-((*S*)-2-(3-(4-Chloro-2-methylphenyl)ureido)-3-phenylpropanamido)-*N*-methoxy-*N*-methyl-4-phenylbutanamide (22) Eluent mixture = light petroleum/EtOAc (1:1); R_f = 0.22 light petroleum/EtOAc (1:1); Yield = 82%; Consistency = white powder; ^{1}H NMR (500 MHz, $CDCl_3$): δ = 1.79-1.92 (m, 1H), 1.89 (s, 3H), 1.94-2.05 (m, 1H), 2.46-2.54 (m, 1H), 2.57-2.65 (m, 1H), 2.86 (dd, J = 13.9, 8.7 Hz, 1H), 3.06-3.17 (m, 1H), 3.12 (s, 3H), 3.55 (s, 3H), 4.80 (dd, J = 13.6, 7.8 Hz, 1H), 4.87-4.95 (m, 1H), 6.41 (d, J = 5.4 Hz, 1H), 6.90-6.99 (m, 2H), 7.05 (d, J = 7.2 Hz, 2H), 7.08-7.21 (m, 9H), 7.28 (d, J = 8.5 Hz, 1H), 7.53 (d, J = 6.3 Hz, 1H). ^{13}C NMR (126 MHz, $CDCl_3$): δ = 17.63, 31.71, 32.26, 33.80, 38.95, 49.46, 55.38, 61.56, 124.86, 126.13, 126.63, 126.94, 128.43, 128.52, 128.56, 129.39, 129.49, 130.25, 132.12, 135.15, 136.88, 140.82, 155.99, 172.18, 173.23.

(*S*)-2-((*S*)-2-(3-(4-Chloro-2-(trifluoromethyl)phenyl)ureido)-3-phenylpropanamido)-*N*-methoxy-*N*-methyl-4-phenylbutanamide (23) Eluent mixture = light petroleum/EtOAc (3:2); R_f = 0.32 light petroleum/EtOAc (3:2); Yield = 21%; Consistency = white powder; ^{1}H NMR (500 MHz, $CDCl_3$): δ = 1.79-1.90 (m, 1H), 1.96-2.06 (m, 1H), 2.49-2.59 (m, 1H), 2.62-2.71 (m, 1H), 2.89 (dd, J = 13.8, 8.4 Hz, 1H), 3.08 (dd, J = 13.9, 5.0 Hz, 1H), 3.16 (s, 3H), 3.62 (s, 3H), 4.80 (dd, J = 13.4, 7.5 Hz, 1H), 5.01-5.09 (m, 1H), 6.99 (d, J = 7.9 Hz, 1H), 7.06 (d, J = 7.2 Hz, 2H), 7.09-7.27 (m, 10H), 7.41 (bs, 2H), 7.68 (d, J = 8.9 Hz, 1H). ^{13}C NMR (126 MHz, $CDCl_3$): δ = 29.83, 31.77, 32.28, 33.96, 39.06, 49.59, 55.42, 61.67, 121.34 (q, J = 29.4 Hz), 123.33 (q, J = 273.0 Hz), 125.89 (q, J = 10.6, Hz), 126.10, 126.27, 126.91, 128.44, 128.48, 128.52, 129.43, 132.49, 135.32 (q, J = 1.6 Hz), 136.75, 140.99, 154.69, 172.29, 173.07.

(*S*)-*N*-Methoxy-*N*-methyl-2-((*S*)-2-(3-(naphthalen-1-yl)ureido)-3-phenylpropanamido)-4-phenylbutanamide (24) Eluent mixture = light petroleum/EtOAc (1:1); R_f = 0.26 light petroleum/EtOAc (1:1); Yield = 88%; Consistency = white powder; ^{1}H NMR (500 MHz, $CDCl_3$): δ = 1.74-1.82 (m, 1H), 1.89-1.98 (m, 1H), 2.39-2.47 (m, 1H), 2.49-2.56 (m, 1H), 2.97 (dd, J = 13.9, 7.6 Hz, 1H), 3.01-3.10 (m, 1H), 3.04 (s, 3H), 3.37 (s, 3H), 4.79-4.90 (m, 2H), 6.07 (bs, 1H), 6.97-7.16 (m, 10H), 7.24-7.34 (m, 4H), 7.35-7.42 (m, 1H), 7.46-7.50 (m, 1H), 7.58-7.65 (m, 1H), 7.78 (d, J = 7.9 Hz, 1H), 7.80 (bs, 1H), 7.78 (d, J = 7.9 Hz, 1H). ^{13}C NMR (126 MHz, $CDCl_3$): δ = 31.54, 32.12, 33.95, 38.66, 49.14, 55.29, 61.38, 122.08, 122.28, 125.88, 125.98, 126.21, 126.47, 126.81, 128.34, 128.40, 128.52, 128.54, 128.82, 128.89, 129.42, 133.40, 134.40, 136.76, 141.01, 156.69, 171.99, 172.23.

(*S*)-*N*-((*S*)-1-(methoxy(methyl)amino)-1-oxo-4-phenylbutan-2-yl)-4-methyl-2-(3-(*p*-tolyl)ureido)pentanamide (29) Eluent mixture = light petroleum/EtOAc (1:1); R_f = 0.29 light petroleum/EtOAc (1:1); Yield = 43%; Consistency = white powder; ^{1}H NMR (500 MHz, $CDCl_3$): δ = 0.93 (t, J = 6.4 Hz, 6H), 1.47-1.56 (m, 1H), 1.58-167 (m, 1H), 1.68-1.78 (m, 1H), 1.86-1.98 (m ,1H), 2.01-2.12 (m, 1H), 2.24 (s, 3H), 2.55-.274 (m, 2H), 3.15 (s, 3H), 3.59 (s, 3H), 4.48 (td, J = 9.2, 5.5 Hz, 1H), 4.91-5.02 (m, 1H), 5.92 (d, J = 8.2 Hz, 1H), 6.96 (d, J = 8.1 Hz, 2H),7.04-7.15 (m, 6H), 7.16-7.23 (m, 3H). ^{1}H NMR (400 MHz, $CDCl_3$): δ = 20.88, 22.19, 23.17, 24.96, 31.69, 32.32, 34.07, 41.95, 49.48, 52.88, 61.64, 120.94, 126.11, 128.47, 128.67, 129.64, 133.01, 136.14, 141.02, 155.95, 172.29, 174.12.

(*S*)-2-(3-(4-Chloro-2-methylphenyl)ureido)-*N*-((*S*)-1-(methoxy(methyl)amino)-1-oxo-4-phenylbutan-2-yl)-4-methylpentanamide (30) Eluent mixture = light petroleum/EtOAc (2:3); R_f = 0.35 light petroleum/EtOAc (2:3); Yield = 45%; Consistency = white powder; ^{1}H NMR (500 MHz, $CDCl_3$): δ = 0.83-0.89 (m, 6H), 1.35-1.47 (m, 1H), 1.48-1.67 (m, 2H), 1.79-1.90 (m, 1H), 1.95-2.03 (m, 1H), 2.06 (s, 3H), 2.48-2.68 (m, 2H), 3.08 (s, 3H), 3.53 (s, 3H), 4.32-4.41 (m, 1H), 4.81-4.92 (m, 1H), 5.64 (d, J = 8.0 Hz, 1H), 6.56 (s, 1H), 6.81 (d, J = 8.0 Hz, 1H), 6.98-7.04 (m, 2H), 7.05-7.11 (m, 3H), 7.13-7.19 (m, 2H), 7.40 (d, J = 8.5 Hz, 1H). ^{13}C NMR (126 MHz, $CDCl_3$): δ = 17.86, 22.11, 23.17, 25.02, 31.72, 32.36, 34.13, 41.90, 49.44, 53.01, 61.59, 125.36, 126.24, 127.01, 127.67, 128.53, 128.66, 130.60, 132.74, 135.03, 141.00, 155.84, 172.22, 173.65.

1.1.2 General procedure for the synthesis of final compounds 1-9, 25, and 26.

In a round-bottom flask, the appropriate intermediate **16-24, 29**, or **30** (1 eq.) was solubilized in dry THF (10 mL/mmol), cooled down to 0°C with an ice-bath and vigorously stirred. After that, 1 eq. of $LiAlH_4$ was added each 30 min until the TLC monitoring did not show the presence of starting material (usually, 2 or 3 eq. of $LiAlH_4$ were added). The presence of aldehyde was detected using the 2,4-dinitrophenylhydrazine TLC stain. Subsequently, the excess of $LiAlH_4$ was quenched with a few drops of 1 M $KHSO_4$ and the ice-bath was removed. The suspension was moved in a separatory funnel, DCM was added, the two phases were separated and the organic phase was further extracted with DCM (x3). The merged organic phases were washed with $NaHCO_3$ sat. sol. (x2), 1 M $KHSO_4$ (x1) and brine (x1), dried over Na_2SO_4 and concentrated in *vacuo*. The obtained residue was used for the next step without further purification. In a round bottom flask, the aldehyde was solubilized in DCM (5 mL/mmol) and 1-(triphenylphosphoranylidene)-2-propanone (1 eq.) was added. The reaction was stirred at rt for 2h. After this time, the solvent was removed in *vacuo* and the desired product was purified by column chromatography using the appropriate eluent (see details in the section below).

(*S*)-*N*-((*S*,*E*)-6-Oxo-1-phenylhept-4-en-3-yl)-3-phenyl-2-(3-phenylureido)propenamide (1) Eluent mixture = light petroleum/EtOAc (2:3); R_f = 0.45 light petroleum/EtOAc (2:3); Yield = 52%; Consistency = white powder. ^{1}H NMR (500 MHz, DMSO): δ = 1.69-1.79 (m, 1H), 1.82-1.92 (m, 1H), 2.15 (s, 3H), 2.50-2.67 (m, 2H), 2.85 (dd, *J* = 13.5, 7.9 Hz, 1H), 2.97 (dd, *J* = 13.6, 6.1 Hz, 1H), 4.37-4.45 (m, 1H), 4.53 (dd, *J* = 14.5, 6.9 Hz, 1H), 5.87 (d, *J* = 16.1 Hz, 1H), 6.37 (d, *J* = 8.0 Hz, 1H), 6.67 (dd, *J* = 16.1, 5.2 Hz, 1H), 6.86 (t, *J* = 7.3 Hz, 1H), 7.09-7.29 (m, 12H), 7.34 (d, *J* = 8.0 Hz, 2H), 8.31 (d, *J* = 8.1 Hz, 1H), 8.63 (s, 1H); ^{13}C NMR (126 MHz, DMSO) δ = 198.40, 171.74, 155.04, 148.21, 141.73, 140.66, 137.79, 129.86, 129.58, 129.23, 128.96, 128.69, 128.53, 126.99, 126.37, 121.58, 118.09, 54.68, 49.42, 39.11, 35.58, 31.83, 27.56. Elemental analysis calcd for $C_{29}H_{31}N_3O_3$: C, 74.18; H, 6.65; N, 8.95; found: C, 73.96; H, 6.57; N, 73.81.

(*S*)-2-(3-(4-Methoxyphenyl)ureido)-*N*-((*S*,*E*)-6-oxo-1-phenylhept-4-en-3-yl)-3-phenylpropanamide (2) Eluent mixture = light petroleum/EtOAc (1:1); R_f = 0.36 light petroleum/EtOAc (1:1); Yield = 36%; Consistency = white powder. ^{1}H NMR (500 MHz, DMSO): δ = 1.69-1.80 (m, 1H), 1.83-1.93 (m, 1H), 2.17 (s, 3H), 2.53-2.68 (m, 2H), 2.86 (dd, *J* = 13.4, 7.8 Hz, 1H), 2.98 (dd, *J* = 13.6, 5.8 Hz, 1H), 3.68 (s, 3H), 4.38-4.46 (m, 1H), 4.53 (dd, *J* = 14.4, 7.1 Hz, 1H), 5.88 (d, *J* = 16.1 Hz, 1H), 6.29 (d, *J* = 8.1 Hz, 1H), 6.69 (dd, *J* = 16.1, 5.2 Hz, 1H), 6.79 (d, *J* = 7.3 Hz, 2H), 7.10-7.32 (m, 12H), 8.30 (d, *J* = 8.0 Hz, 1H), 8.74 (s, 1H), ^{13}C NMR (126 MHz, DMSO): δ = 198.38, 171.84, 155.26, 154.40, 148.21, 141.74, 137.87, 133.82, 129.85, 129.58, 128.94, 128.80, 128.71, 128.40, 126.38, 119.75, 114.35, 55.66, 55.48, 49.41, 39.15, 35.58, 31.82, 27.41. Elemental analysis calcd for $C_{30}H_{33}N_3O_4$: C, 72.12; H, 6.66; N, 8.41; found: C, 72.34; H, 6.84; N, 8.30.

(*S*)-*N*-((*S*,*E*)-6-Oxo-1-phenylhept-4-en-3-yl)-3-phenyl-2-(3-(p-tolyl)ureido)propanamide (3) Eluent mixture = light petroleum/EtOAc (2:3); R_f = 0.44 light petroleum/EtOAc (2:3); Yield = 73%; Consistency = white powder. ^{1}H NMR (500 MHz, DMSO): δ = 1.71-1.80 (m ,1H), 1.83-1.93 (m, 1H), 2.17 (s, 3H), 2.20 (s,

3H), 2.54-2.67 (m, 2H), 2.86 (dd, *J* = 13.6, 7.7 Hz, 1H), 2.98 (dd, *J* = 13.8, 6.2 Hz, 1H), 4.38-4.46 (m ,1H), 4.53 (dd, *J* = 14.5, 7.5 Hz, 1H), 5.88 (d, *J* = 16.1 Hz, 1H), 6.33 (d, *J* = 8.2 Hz, 1H), 6.69 (dd, *J* = 16.1, 5.2 Hz, 1H), 7.00 (d, *J* = 8.4 Hz, 2H), 7.11-7.31 (m, 12H), 8.31 (d, *J* = 8.3 Hz, 1H), 8.53 (s, 1H); ^{13}C NMR (126 MHz, DMSO) δ = 198.62, 179.46, 171.79, 155.04, 147.98, 141.50, 137.75, 137.55, 130.42, 129.69, 129.44, 129.29, 128.80, 128.61, 128.37, 126.91, 126.26, 118.16, 54.63, 49.26, 40.24, 35.35, 31.64, 27.36, 20.61. Elemental analysis calcd for $C_{30}H_{33}N_3O_3$: C, 74.51; H, 6.88; N, 8.69; found: C, 74.36; H, 7.03; N, 8.44.

(*S*)-2-(3-(4-Fluorophenyl)ureido)-*N*-((*S*,*E*)-6-oxo-1-phenylhept-4-en-3-yl)-3-phenylpropanamide (4)
Eluent mixture = light petroleum/EtOAc (1:1); R_f = 0.28 light petroleum/EtOAc (1:1); Yield = 55%; Consistency = white powder. ^{1}H NMR (500 MHz, DMSO): δ = 1.71-1.80 (m, 1H), 1.84-1.94 (m, 1H), 2.17 (s, 3H), 2.13-2.20 (m, 2H), 2.87 (dd, *J* = 13.6, 7.8 Hz, 1H), 2.99 (dd, *J* = 13.7, 6.1 Hz, 1H), 4.38-4.46 (m, 1H), 4.54 (dd, *J* = 14.2, 7.9 Hz, 1H), 5.89 (dd, *J* = 16.1, 1.6 Hz, 1H), 6.41 (d, *J* = 8.3 Hz, 1H), 6.69 (dd, *J* = 16.1, 5.2 Hz, 1H), 6.98-7.08 (m, 2H), 7.13-7.29 (m, 10H), 7.33-7.40 (m, 2H), 8.32 (d, *J* = 8.3 Hz, 1H), 8.73 (s, 1H); ^{13}C NMR (126 MHz, DMSO): δ = 197.93, 171.26, 156.77 (d, *J* = 264.5 Hz), 154.64, 147.73, 141.27, 137.34, 136.59 (d, *J* = 2.3 Hz), 129.39, 129.14, 128.48, 128.24, 128.09, 126.53, 125.92, 119.17 (d, *J* = 7.0 Hz), 115.02 (d, *J* = 17.0 Hz), 54.24, 48.97, 38.64, 35.15, 31.37, 27.02. Elemental analysis calcd for $C_{29}H_{30}FN_3O_3$: C, 71.44; H, 6.20; N, 8.62; found: C, 71.24; H, 5.96; N, 8.55.

(*S*)-2-(3-(2,6-Difluorophenyl)ureido)-*N*-((*S*,*E*)-6-oxo-1-phenylhept-4-en-3-yl)-3-phenylpropanamide (5)
Eluent mixture = light petroleum/EtOAc (1:1); R_f = 0.25 light petroleum/EtOAc (1:1); Yield = 29%; Consistency = white powder. ^{1}H NMR (500 MHz, DMSO): δ = 1.70-1.79 (m, 1H), 1.83-1.92 (m, 1H), 2.17 (s, 3H), 2.54-2.65 (m, 2H), 2.88 (dd, *J* = 13.4, 7.4 Hz, 1H), 2.99 (dd, *J* = 13.6, 6.5 Hz, 1H), 4.37-4.54 (m, 1H), 4.52 (dd, *J* = 14.7, 7.3 Hz, 1H), 5.87 (d, *J* = 16.0 Hz, 1H), 6.64 (d, *J* = 9.0 Hz, 1H), 6.67 (dd, *J* = 16.3, 5.5 Hz, 1H), 7.07 (t, *J* = 8.0 Hz, 2H), 7.12-7.30 (m, 11H), 8.06 (s, 1H), 8.30 (d, *J* = 8.2 Hz, 1H); ^{13}C NMR (126 MHz, DMSO) δ = 198.56, 171.07, 156.00 (dd, *J* = 212.3, 21.9 Hz),154.39, 147.66, 141.29, 137.26, 129.47, 129.24, 128.55, 128.31, 128.09, 126.97, 126.53 (t, *J* = 4.3 Hz), 126.38, 118.84 (t, *J* = 17.7 Hz), 114.23 (dd, *J* = 36.6, 5.2 Hz), 54.56, 48.94, 38.65, 35.17, 31.35, 27.13. Elemental analysis calcd for $C_{29}H_{29}F_2N_3O_3$: C, 68.90; H, 5.78; N, 8.31; found: C, 69.16; H, 5.57; N: 8.14.

(*S*)-2-(3-(4-Chlorophenyl)ureido)-*N*-((*S*,*E*)-6-oxo-1-phenylhept-4-en-3-yl)-3-phenylpropanamide (6)
Eluent mixture = light petroleum/EtOAc (1:1); R_f = 0.32 light petroleum/EtOAc (1:1); Yield = 64%; Consistency = white powder. ^{1}H NMR (500 MHz, DMSO): δ = 1.71-1.81 (m, 1H), 1.84-1.94 (m, 1H), 2.18 (s, 3H), 2.52-2.68 (m, 2H), 2.88 (dd, *J* = 13.5, 7.7 Hz, 1H), 3.00 (dd, *J* = 13.6, 6.0 Hz, 1H), 4.39-4.47 (m, 1H), 4.55 (dd, *J* = 14.6, 7.1 Hz, 1H), 5.89 (d, *J* = 16.1 Hz, 1H), 6.43 (d, *J* = 8.0 Hz, 1H), 6.70 (dd, *J* = 16.1, 5.2 Hz, 1H), 7.10-7.32 (m, 12H), 7.39 (d, *J* = 7.3 Hz, 2H), 8.34 (d, *J* = 8.2 Hz, 1H), 8.81 (s, 1H); ^{13}C NMR (126 MHz, DMSO) δ = 197.94, 171.15, 154.43, 147.71, 141.26, 139.21, 137.27, 129.37, 129.17, 128.45, 128.27, 128.13, 126.51, 125.89, 124.60, 119.07, 54.19, 49.01, 38.65, 35.13, 31.38, 30.74, 27.08. Elemental analysis calcd for $C_{29}H_{30}ClN_3O_3$: C, 69.11; H, 6.00; N, 8.34; found: C, 68.91; H, 6.17; N, 8.22.

(*S*)-2-(3-(4-Chloro-2-methylphenyl)ureido)-*N*-((*S*,*E*)-6-oxo-1-phenylhept-4-en-3-yl)-3-phenylpropanamide (7) Eluent mixture = light petroleum/EtOAc (1:1); R_f = 0.43 light petroleum/EtOAc (1:1); Yield = 53%; Consistency = white powder. ^{1}H NMR (500 MHz, DMSO): δ = 1.69-1.78 (m, 1H), 1.82-1.90 (m, 1H), 2.14 (s, 3H), 2.15 (s, 3H), 2.85 (dd, J = 13.6, 7.5 Hz, 1H), 2.97 (dd, J = 13.7, 6.4 Hz, 1H), 4.36-4.46 (m, 1H), 4.54 (dd, J = 14.7, 7.5 Hz, 1H), 5.84 (d, J = 16.1 Hz, 1H), 6.66 (dd, J = 16.1, 5.3 Hz, 1H), 6.97 (d, J = 8.3 Hz, 1H), 7.08 (dd, J = 8.8, 2.5 Hz, 1H), 7.11-7.27 (m, 8H), 7.83 (d, J = 8,8 Hz, 1H), 7.92 (s, 1H), 8.32 (d, J = 8.3 Hz, 1H); ^{13}C NMR (126 MHz, DMSO) δ = 198.04, 171.23, 154.65, 147.80, 141.30, 137.38, 137.12, 129.67, 129.27, 129.06, 128.83, 128.57, 128.45, 128.27, 128.13, 127.98, 126.61, 125.85, 121.52, 54.42, 49.07, 38.87, 35.19, 31.44, 27.14, 17.78. Elemental analysis calcd for $C_{30}H_{32}ClN_3O_3$: C, 69.55; H, 6.23; N, 8.11; found: C; 69.47; H, 5.98; N, 8.28.

(*S*)-2-(3-(4-Chloro-2-(trifluoromethyl)phenyl)ureido)-*N*-((*S*,*E*)-6-oxo-1-phenylhept-4-en-3-yl)-3-phenylpropanamide (8) Eluent mixture = light petroleum/EtOAc (3:2); R_f = 0.46 light petroleum/EtOAc (3:2); Yield = 51%; Consistency = white powder. ^{1}H NMR (500 MHz, DMSO): δ = 1.70-1.80 (m, 1H), 1.84-1.93 (m, 1H), 2.17 (s, 3H), 2.54-2.68 (m, 2H), 2.86 (dd, J = 13.6, 7.6 Hz, 1H), 3.00 (dd, J = 13.6, 6.5 Hz, 1H), 4.38-4.48 (m, 1H), 4.56 (dd, J = 14.5, 7.6 Hz, 1H), 5.87 (d, J = 16.0 Hz, 1H), 6.68 (dd, J = 16.1, 5.2 Hz, 1H), 7.13-7.29 (m, 10H), 7.51 (d, J = 8.1 Hz, 1H), 7.60 (dd, J = 9.0, 2.4 Hz, 1H), 7.65 (d, J = 2.5 Hz, 1H), 7.93 (d, J = 9.0 Hz, 1H), 8.13 (s, 1H), 8.36 (d, J = 8.3 Hz, 1H);); ^{13}C NMR (126 MHz, DMSO) δ = 198.07, 170.92, 154.26, 147.74, 141.29, 137.24, 136.19 (q, J = 1.9 Hz), 134.17, 132.75 (q, J = 1.4 Hz), 132.47, 129.49, 129.19, 128.53, 128.27, 128.12 (q, J = 2.0 Hz), 127.95 (q, J = 7.2, 3.8 Hz), 126.50, 126.17 (q, J = 38.0 Hz), 125.56 (q, J = 41.5 Hz), 120.98 (d, J = 256.7 Hz), 54.60, 49.01, 38.78, 35.18, 31.42, 27.13. Elemental analysis calcd for $C_{30}H_{29}ClF_3N_3O_3$: C, 62.99; H, 5.11; N, 7.35; found: C, 63.13; H, 4.91; N, 7.46.

(*S*)-2-(3-(Naphthalen-1-yl)ureido)-*N*-((*S*,*E*)-6-oxo-1-phenylhept-4-en-3-yl)-3-phenylpropanamide (9) Eluent mixture = light petroleum/EtOAc (1:1); R_f = 0.24 light petroleum/EtOAc (1:1); Yield = 47%; Consistency = white powder. ^{1}H NMR (500 MHz, DMSO): δ = 1.72-1.82 (m, 1H), 1.85-1.94 (m, 1H), 2.17 (s, 3H), 2.54-2.70 (m, 2H), 2.92 (dd, J = 13.6, 7.7 Hz, 1H), 3.04 (dd, J = 13.4, 6.4 Hz, 1H), 4.59-4.65 (m,1H), 4.62 (dd, J = 14.7, 7.5 Hz, 1H), 5.89 (d, J = 16.0 Hz, 1H), 6.70 (dd, J = 16.0, 5.3 Hz, 1H), 7.01 (d, J = 8.1 Hz, 1H), 7.11-7.32 (m, 9H), 7.39 (t, J = 7.9 Hz, 1H), 7.48-7.58 (m, 3H), 7.59-7.66 (m, 1H), 7.88 (d, J = 7.9 Hz, 1H), 7.99 (d, J = 7.6 Hz, 1H), 8.09 (d, J = 8.3 Hz, 1H), 8.37 (d, J = 8.0 Hz, 1H), 8.73 (s, 1H); ^{13}C NMR (126 MHz, DMSO) δ = 197.98, 171.28, 155.01, 147.75, 141.29, 137.40, 134.94, 134.83, 133.68, 131.65, 131.39, 129.47, 129.21, 129.07, 128.84, 128.50, 128.38, 128.26, 128.12, 127.99, 125.93, 125.71, 125.57, 125.38, 122.26, 121.37, 116.34, 54.42, 49.02, 38.87, 35.17, 31.41, 27.13. Elemental analysis calcd for $C_{33}H_{33}N_3O_3$: C, 76.28; H, 6.40; N, 8.09; found: C, 76.43; H, 6.78; N, 7.94.

(*S*)-4-Methyl-*N*-((*S*,*E*)-6-oxo-1-phenylhept-4-en-3-yl)-2-(3-(p-tolyl)ureido)pentanamide (25) Eluent mixture = light petroleum/EtOAc (1:1); R_f = 0.43 light petroleum/EtOAc (1:1); Yield = 70%; Consistency = white powder. ^{1}H NMR (500 MHz, DMSO): δ = 0.92 (d, J = 6.6 Hz, 3H), 0.94 (d, J = 6.7 Hz, 3H), 1.52 – 1.39 (m, 2H), 1.58 – 1.70 (m, 1H), 1.84 – 1.73 (m, 1H), 1.98 – 1.86 (m, 1H), 2.18 (s, 3H), 2.20 (s, 3H), 2.72

– 2.54 (m, 2H), 4.30 (td, J = 8.5, 6.3 Hz, 1H), 4.49 – 4.39 (m, 1H), 5.99 (dd, J = 16.1, 1.7 Hz, 1H), 6.28 (d, J = 8.4 Hz, 1H), 6.80 (dd, J = 16.1, 5.0 Hz, 1H), 7.01 (d, J = 8.2 Hz, 2H), 7.29 – 7.11 (m, 7H), 8.33 (d, J = 8.3 Hz, 1H), 8.47 (s, 1H). ^{13}C NMR (126 MHz, DMSO) δ = 20.26, 22.04, 22.94, 24.34, 26.86, 31.43, 35.02, 42.08, 48.93, 51.47, 117.57, 125.78, 128.22, 128.29, 128.37, 129.04, 129.80, 137.74, 141.28, 148.15, 154.81, 172.52, 198.00. Elemental analysis calcd for $C_{27}H_{35}N_3O_3$: C, 72.13; H, 7.85; N, 9.35; found: C, 71.90; H, 8.11; N, 9.16.

(*S*)-2-(3-(4-chloro-2-methylphenyl)ureido)-4-methyl-*N*-((*S*,*E*)-6-oxo-1-phenylhept-4-en-3-yl)pentanamide (26) Eluent mixture = light petroleum/EtOAc (1:1); R_f = 0.54 light petroleum/EtOAc (1:1); Yield = 58%; Consistency = white powder. ^{1}H NMR (500 MHz, DMSO): δ = 0.92 (d, J = 6.5 Hz, 3H), 0.95 (d, J = 6.6 Hz, 3H), 1.54 – 1.41 (m, 2H), 1.70 – 1.59 (m, 1H), 1.84 – 1.73 (m, 1H), 1.96 – 1.86 (m, 1H), 2.18 (s, 3H), 2.19 (s, 3H), 2.72 – 2.53 (m, 2H), 4.37 – 4.26 (m, 1H), 4.50 – 4.39 (m, 1H), 6.00 (dd, J = 16.1, 1.7 Hz, 1H), 6.80 (dd, J = 16.1, 5.0 Hz, 1H), 6.92 (d, J = 8.3 Hz, 1H), 7.10 (dd, J = 8.8, 2.5 Hz, 1H), 7.13 – 7.22 (m, 4H), 7.22 – 7.28 (m, 2H), 7.88 (s, J = 9.0 Hz, 1H), 7.94 (d, J = 8.8 Hz, 1H), 8.36 (d, J = 8.3 Hz, 1H). ^{13}C NMR (126 MHz, DMSO) δ = 17.56, 22.03, 22.87, 24.30, 26.80, 31.41, 35.03, 42.05, 48.92, 51.59, 121.11, 125.08, 125.66, 125.75, 128.18, 128.31, 128.55, 129.06, 129.42, 137.18, 141.25, 148.00, 154.70, 172.34, 197.93. Elemental analysis calcd for $C_{27}H_{34}ClN_3O_3$: C, 67.00; H, 7.08; N, 8.68; found: C, 67.19; H, 6.83; N, 8.42.

Biological evaluation

In all the manipulations regarding the biological evaluation, no unexpected or unusually high safety hazards were encountered.

Enzyme assays towards rhodesain.

The biological activity against rhodesain was evaluated by fluorescence-based assays and all tested compounds were diluted in DMSO. Rhodesain was recombinantly expressed as previously reported[2,3] and Cbz-Phe-Arg-AMC (10 μM) was used as the fluorogenic substrate. In the preliminary screening at 0.1 μM, the substrate hydrolysis was determined over a period of 10 min at rt and fluorescence intensity was measured every 30 seconds, whereas the kinetic parameters after the dilutions were determined over a period of 30 min. The assay buffer (pH 5.5) contains 50 mM of sodium acetate, 5 mM of EDTA, 200 mM of NaCl, and 0.005% of Brij 35. Enzyme buffer (pH 5.5) contains 5 mM of DTT and the same chemicals used for assay buffer, with exception of Brij 35. Each independent assay was carried-out twice in duplicate using 96-well-plates in a final volume of 200 μL. Infinity 200 PRO microplate (Tecan, Männedorf, Switzerland) was employed as the fluorescence reader, and a 380 nm excitation filter and a 460 nm emission filter were used. The first-order inactivation rate costants (k_{obs}) were determined analyzing the progress curves by nonlinear regression analysis using the equation 1 shown in the **Chart S1**.[4] The obtained k_{obs} values were fitted against the inhibito concentrations to the hyperbolic equation 2, providing the k_{inact} and K_{iapp} values. The latter were corrected to to zero substrate concentration by the term (1+[S]/K_m) in equation 3. The K_m value used to correct K_{iapp} values was determined to 0.9 μM.[5] K_i and k_{inac} values were calculated by nonlinear regression analyses using GraFit.[6] The second-order rate constants $k_{2nd} = k_{inact}/K_i$ were easily determined from the individual values.

1) $$F = A \cdot (1^{-(-k_{obs}t)}) + B$$

2) $$k_{obs} = \frac{k_{inact} \cdot [I]}{(K_{iapp} + [I])}$$

3) $$K_i = \frac{K_{iapp}}{(1 + [S]/K_m)}$$

Chart S1. Equations 1, 2, and 3.

Enzyme assays towards hCatL.

The biological evaluation towards hCatL was performed following the procedure reported in the literature.[7]

Enzyme assays towards SARS-CoV-2 M^{pro}.

The biological activity of novel Michael acceptors **1-9** against SARS-CoV-2 M^{pro} was carried-out following the previously described protocol.[8]

Antitrypanosomal activity.

EC_{50} values for the antitrypanosomal activity were determined using the ATPlite assay as previously described.[9-11]

Molecular Modeling.

Docking. *In silico* molecular docking calculations were performed employing AutoDock4 (AD4) software.[12] The covalent adduct formation between protein and ligand was modeled according to the covalent docking protocol developed by Bianco et al. called the "flexible side chain method".[13] This technique involves the modification of the residue that plays a major role in the covalent bond formation (i.e., C25) by connecting it to the ligand undergoing the Michael-addition reaction. Consecutively, the altered C25 residue is treated as flexible throughout the docking calculations. To this end, compounds **1-9**, **25**, and **26** were docked. The ligands were constructed using the Maestro suite along with two additional atoms where the alkylation takes place.[14] Principally, the two additional atoms include a carbon and a sulfur atom, to match the corresponding atoms in the C25 residue. The X-ray crystal structure of rhodesain was downloaded from the RCSB PDB database having the PDB: 2P86[15] and further prepared for docking calculations using the protein preparation wizard, part of the Schrodinger suite.[16] The overlay of the ligands to the C25 residue was accomplished using the scripts presented in the AD4 website.[17] The rhodesain receptor grid was considered through mapping the interaction energies of the receptor as well as exploiting the ligand atom types as probes utilizing AutoGrid4. The grid box was constructed with 60Å×60Å×60Å of 0.375Å spacing and the box was positioned on the coordinates of the 2P86 co-crystal ligand as a reference. Further, the docking calculations were performed by treating the modified C25 residue as flexible., Simultaneously, the Lamarckian Genetic Algorithm (LGA) was employed for the docking simulations.[18] 200 individual LGA runs were executed based on the complexity of the ligand and its high number of torsional degrees of freedom. Every individual docking run consists of 20 million energy evaluations based on the Lamarckian genetic algorithm local search (GALS) method. The GALS routine gives out the most effective docking solutions from each generation into the consequent generation of possible solutions by calculating the population of possible docking solutions. According to the Solis and Wets method, a low-frequency local search is applied to the docking trials to make sure that the final solution represents a local minimum. The dockings were accomplished with a population size of 150, and 300 rounds of Solis and Wets local search were applied with a probability of 0.06. To generate new docking trials for the consequent generations and the best individual from each generation propagated over the next generation, a mutation rate of 0.02 and crossover rate of 0.8 were used. The remaining settings were set at their default values. The docking outcomes were clustered based on the root –mean square deviation (solutions differing by less than 3.0Å) between the Cartesian coordinates of the atoms and were ranked based on binding free energy (ΔG_{AD4}). Finally, the **7** best docking pose with the best-predicted ΔG_{AD4} was selected for rhodesain. All the pictures were rendered using UCSF Chimera software.[19]

Molecular Dynamics. The complex predicted for the most active compound (**7**/rhodesain) was further subjected to molecular dynamics simulation using Desmond module of the Schrodinger software. [20,21] As an initial step in the MD pipeline, system builder panel was employed to prepare the (**7**/rhodesain) docked complex system for the MD calculations. The complex was immersed in a parallelepiped box by solvating it with TIP3P water model.[22] The negative -19 charges in the system were neutralized adding 19 Na^+ ions. Further the equilibration of the system was performed using NPT ensemble with default Desmond

parameters (8-steps). The first 7-steps were considered as the short simulations known as the equilibration phase, as the temperature of the system is gradually increased and the solute is restrained partially. Later, the equilibrated system is subjected to 100ns MD final production along with PBC conditions and NPT ensemble. This simulation system was set up with 300K temperature and 1atm pressure using the Martyna−Tobias−Klein barosta[23] and Nose−Hoover chain thermostat.[24] The OPLS force field [25] was used for all the MD simulation steps.

References

1) Chenna, B. C.; Li, L.; Mellott, D. M.; Zhai, X.; Siqueira-Neto, J. L.; Calvet Alvarez, C.; Bernatchez, J. A.; Desormeaux, E.; Alvarez Hernandez, E.; Gomez, J.; McKerrow, J. H.; Cruz-Reyes, J.; Meek, T. D. Peptidomimetic vinyl heterocyclic inhibitors of cruzain effect antitrypanosomal activity. *J Med Chem* **2020**, *63*, 3298-3316.

2) Latorre, A.; Schirmeister, T.; Kesselring, J.; Jung, S.; Johe, P.; Hellmich, U. A.; Heilos, A.; Engels, B.; Krauth-Siegel, R. L.; Dirdjaja, N.; Bou-Iserte, L.; Rodriguez, S.; Gonzalez, F. V. Dipeptidyl nitroalkenes as potent reversible inhibitors of cysteine proteases rhodesain and cruzain. *ACS Med Chem Lett* **2016**, *7*, 1073-1076.

3) Schirmeister, T.; Kesselring, J.; Jung, S.; Schneider, T. H.; Weickert, A.; Becker, J.; Lee, W.; Bamberger, D.; Wich, P. R.; Distler, U.; Tenzer, S.; Johe, P.; Hellmich, U. A.; Engels, B. Quantum chemical-based protocol for the rational design of covalent inhibitors. *J Am Chem Soc* **2016**, *138*, 8332-8335.

4) Tian, W. X.; Tsou, C. L. Determination of the rate constant of enzyme modification by measuring the substrate reaction in the presence of the modifier. *Biochemistry* **1982**, *21*, 1028-1032.

5) Vicik, R.; Hoerr, V.; Glaser, M.; Schultheis, M.; Hansell, E.; McKerrow, J. H.; Holzgrabe, U.; Caffrey, C. R.; Ponte-Sucre, A.; Moll, H.; Stich, A.; Schirmeister, T. Aziridine-2,3-dicarboxylate inhibitors targeting the major cysteine protease of Trypanosoma brucei as lead trypanocidal agents. *Bioorg Med Chem Lett* **2006**, *16*, 2753-2757.

6) GraFit, Version 5.0.1.3; Erithacus Software Ltd.: London **2006**.

7) Ettari, R.; Previti, S.; Cosconati, S.; Kesselring, J.; Schirmeister, T.; Grasso, S.; Zappalà, M. Synthesis and biological evaluation of novel peptidomimetics as rhodesain inhibitors. *J Enzyme Inhib Med Chem* **2016**, *31*, 1184-1191.

8) Amendola, G.; Ettari, R.; Previti, S.; Di Chio, C.; Messere, A.; Di Maro, S.; Hammerschmidt, S. J.; Zimmer, C.; Zimmermann, R. A.; Schirmeister, T.; Zappala, M.; Cosconati, S. Lead discovery of SARS-CoV-2 main protease inhibitors through covalent docking-based virtual screening. *J Chem Inf Model* **2021**, *61*, 2062-2073.

9) Previti, S.; Ettari, R.; Cosconati, S.; Amendola, G.; Chouchene, K.; Wagner, A.; Hellmich, U. A.; Ulrich, K.; Krauth-Siegel, R. L.; Wich, P. R.; Schmid, I.; Schirmeister, T.; Gut, J.; Rosenthal, P. J.; Grasso, S.; Zappalà, M. Development of novel peptide-based Michael acceptors targeting rhodesain and falcipain-2 for the treatment of Neglected Tropical Diseases (NTDs). *J Med Chem* **2017**, *60*, 6911-6923.

10) Wagner, A.; Le, T. A.; Brennich, M.; Klein, P.; Bader, N.; Diehl, E.; Paszek, D.; Weickhmann, A. K.; Dirdjaja, N.; Krauth-Siegel, R. L.; Engels, B.; Opatz, T.; Schindelin, H.; Hellmich, U. A. Inhibitor-induced dimerization of an essential oxidoreductase from african trypanosomes. *Angew Chem Int Ed Engl* **2019**, *58*, 3640-3644.

11) Klein, P.; Barthels, F.; Johe, P.; Wagner, A.; Tenzer, S.; Distler, U.; Le, T. A.; Schmid, P.; Engel, V.; Engels, B.; Hellmich, U. A.; Opatz, T.; Schirmeister, T. Naphthoquinones as covalent reversible inhibitors of cysteine proteases-studies on inhibition mechanism and kinetics. *Molecules* **2020**, *25*, 2064.

12) Morris, G. M.; Huey, R.; Lindstrom, W.; Sanner, M. F.; Belew, R. K.; Goodsell, D. S.; Olson, A. J. AutoDock4 and AutoDockTools4: automated docking with selective receptor flexibility. *J Comput Chem* **2009**, *30*, 2785-2791.

13) Bianco, G.; Forli, S.; Goodsell, D. S.; Olson, A. J. Covalent docking using autodock: two-point attractor and flexible side chain methods. *Protein Sci.* **2016**, *25*, 295-301.

14) Schrödinger Maestro, Release 2020-4: Schrödinger, LLC, New York, 2020.

15) Kerr, I. D.; Wu, P.; Marion-Tsukamaki, R.; Mackey, Z. B.; Brinen, L. S. Crystal Structures of TbCatB and rhodesain, potential chemotherapeutic targets and major cysteine proteases of Trypanosoma brucei. *PLoS Negl Trop Dis* **2010**, *4*, e701.

16) Sastry, G. M.; Adzhigirey, M.; Day, T.; Annabhimoju, R.; Sherman, W. Protein and ligand preparation: parameters, protocols, and influence on virtual screening enrichments. *J Comput Aided Mol Des* **2013**, *27*, 221-234.

17) http://autodock.scripps.edu/.

18) Cosconati, S.; Forli, S.; Perryman, A. L.; Harris, R.; Goodsell, D. S.; Olson, A. J. Virtual screening with AutoDock: theory and practice. *Expert Opin Drug Discov* **2010**, *5*, 597-607.
19) Pettersen, E. F.; Goddard, T. D.; Huang, C. C.; Couch, G. S.; Greenblatt, D. M.; Meng, E. C.; Ferrin, T. E. UCSF Chimera--a visualization system for exploratory research and analysis. *J Comput Chem* **2004**, *25*, 1605-1612.
20) Jiang, W.; Phillips, J. C.; Huang, L.; Fajer, M.; Meng, Y.; Gumbart, J. C.; Luo, Y.; Schulten, K.; Roux, B. Generalized scalable multiple copy algorithms for molecular dynamics simulations in NAMD. *Comput Phys Commun* **2014**, *185*, 908-916.
21) Schrödinger Release 2020-4: Desmond Molecular Dynamics System, D. E. Shaw Research, New York, NY, 2020. Maestro-Desmond Interoperability Tools, Schrödinger, New York, NY, 2020.
22) Mark, P.; Nilsson, L. Structure and dynamics of the TIP3P, SPC, and SPC/E water models at 298 K. *J Phys Chem A* **2001**, *105*, 9954-9960.
23) Martyna, G. J.; Tuckerman, M. E.; Tobias, D. J.; Klein, M. L. Explicit reversible integrators for extended systems dynamics. *Mol Phys* **1996**, *87*, 1117-1157.
24) Hoover, W. G. Canonical dynamics: equilibrium phase-space distributions. *Phys Rev A Gen Phys* **1985**, *31*, 1695-1697.
25) Harder, E.; Damm, W.; Maple, J.; Wu, C.; Reboul, M.; Xiang, J. Y.; Wang, L.; Lupyan, D.; Dahlgren, M. K.; Knight, J. L.; Kaus, J. W.; Cerutti, D. S.; Krilov, G.; Jorgensen, W. L.; Abel, R.; Friesner, R. A. OPLS3: A force field providing broad coverage of drug-like small molecules and proteins. *J Chem Theory Comput* **2016**, *12*, 281-296.

NMR spectra

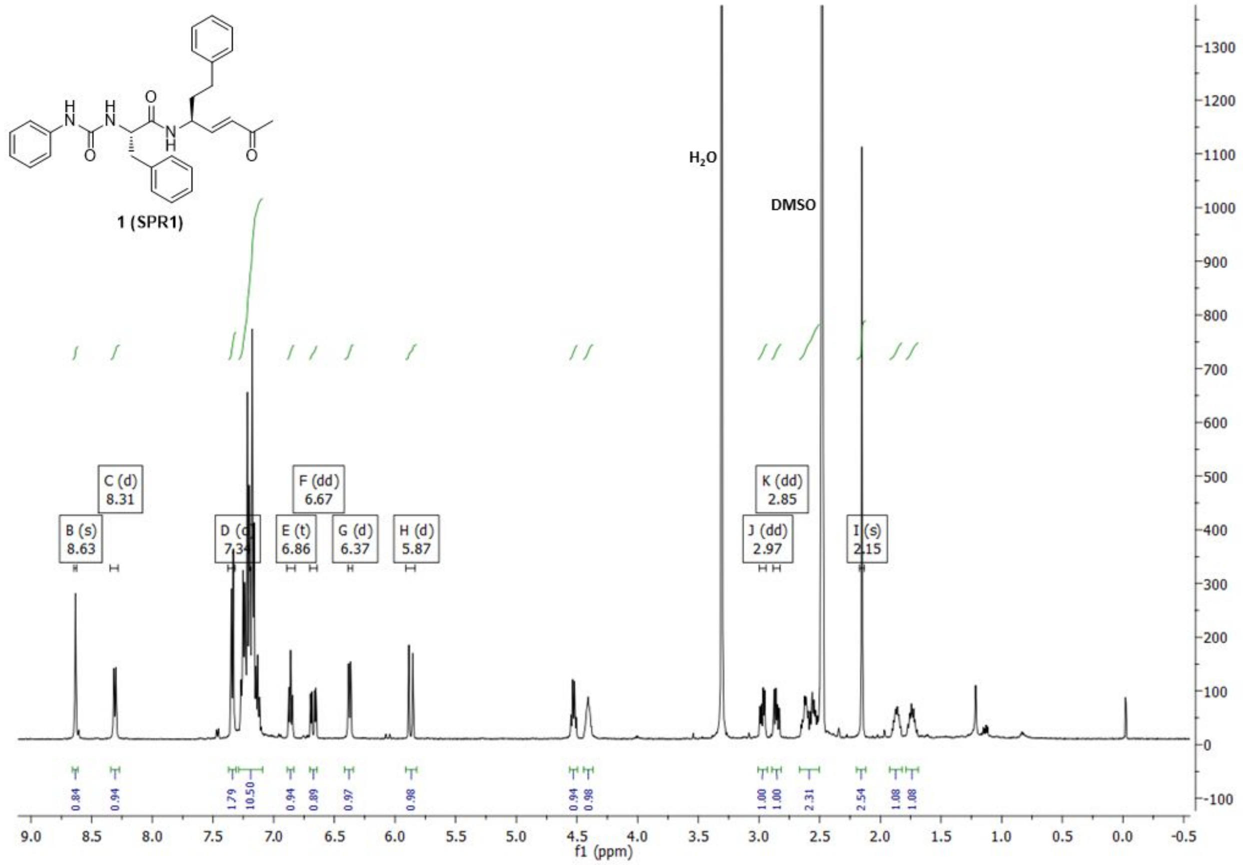

Figure S1. ^{1}H NMR of compound 1.

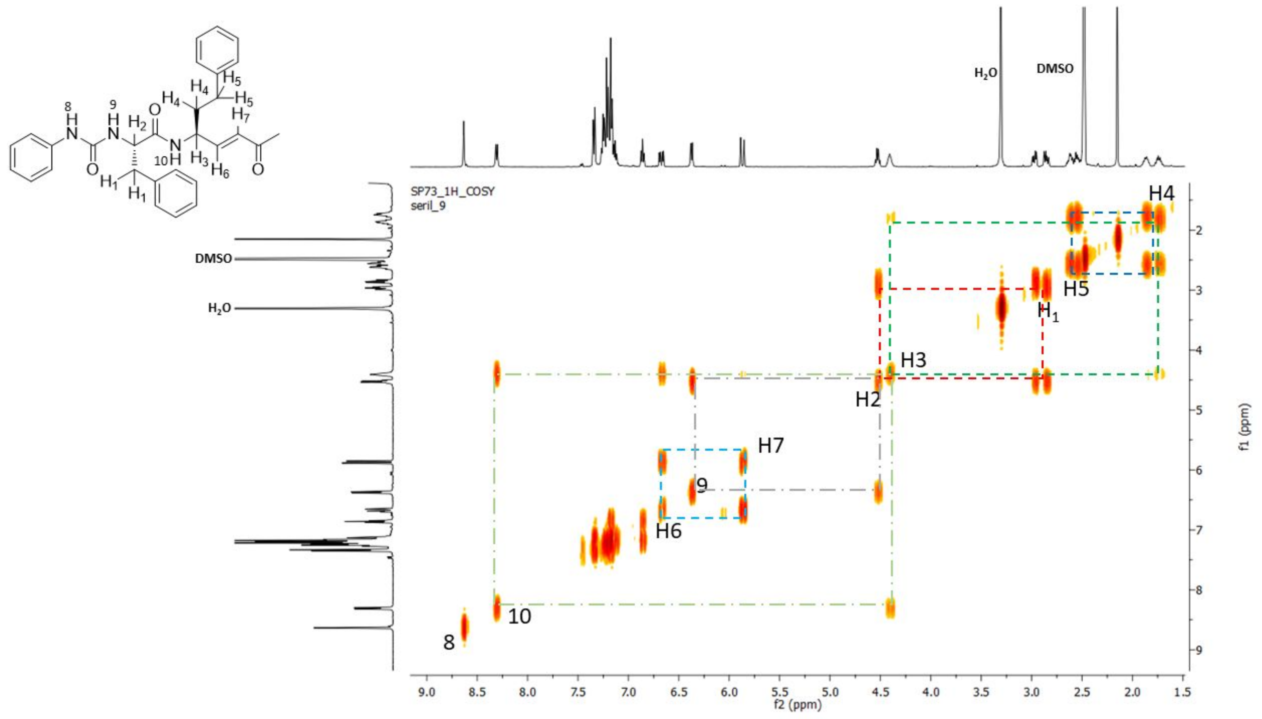

Figure S2. COSY of compound 1.

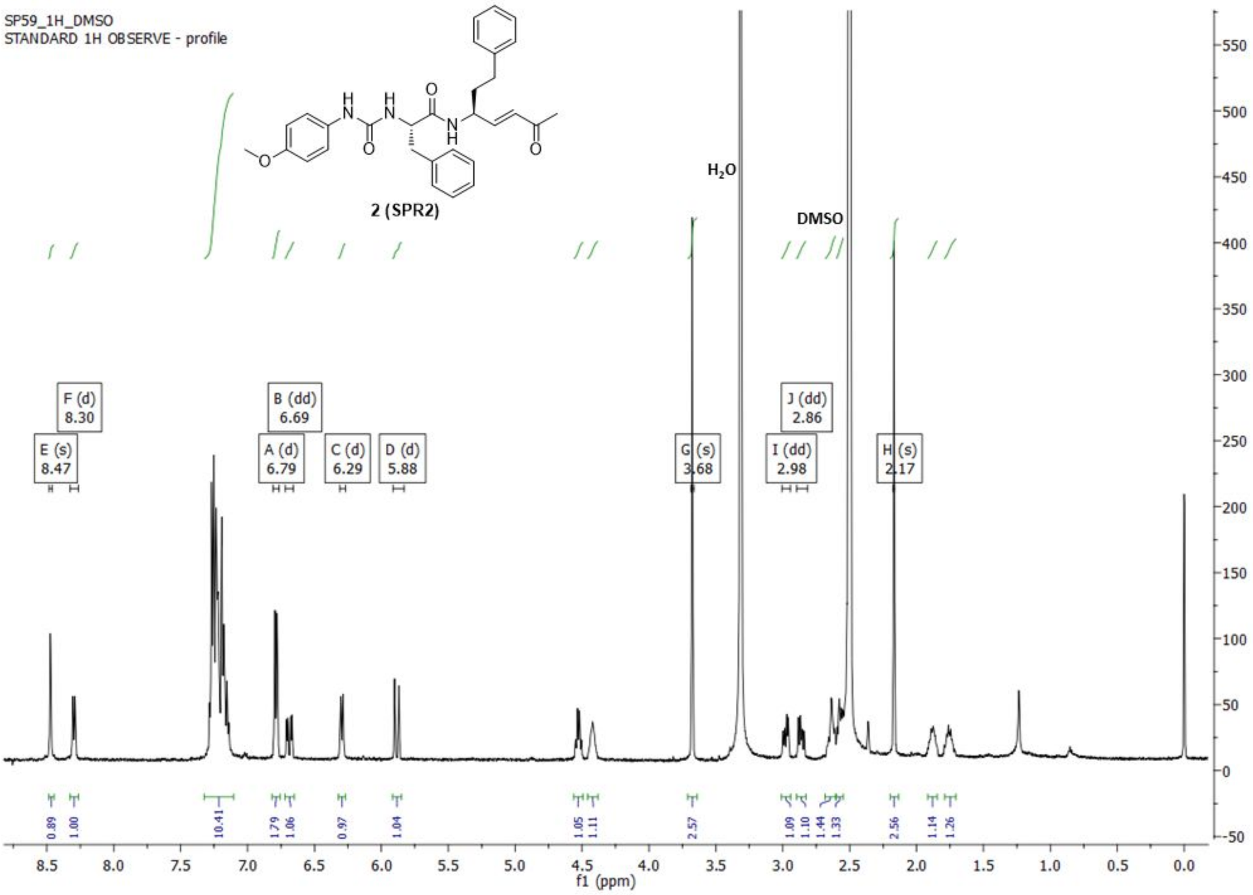

Figure S3. ^{1}H NMR of compound 2.

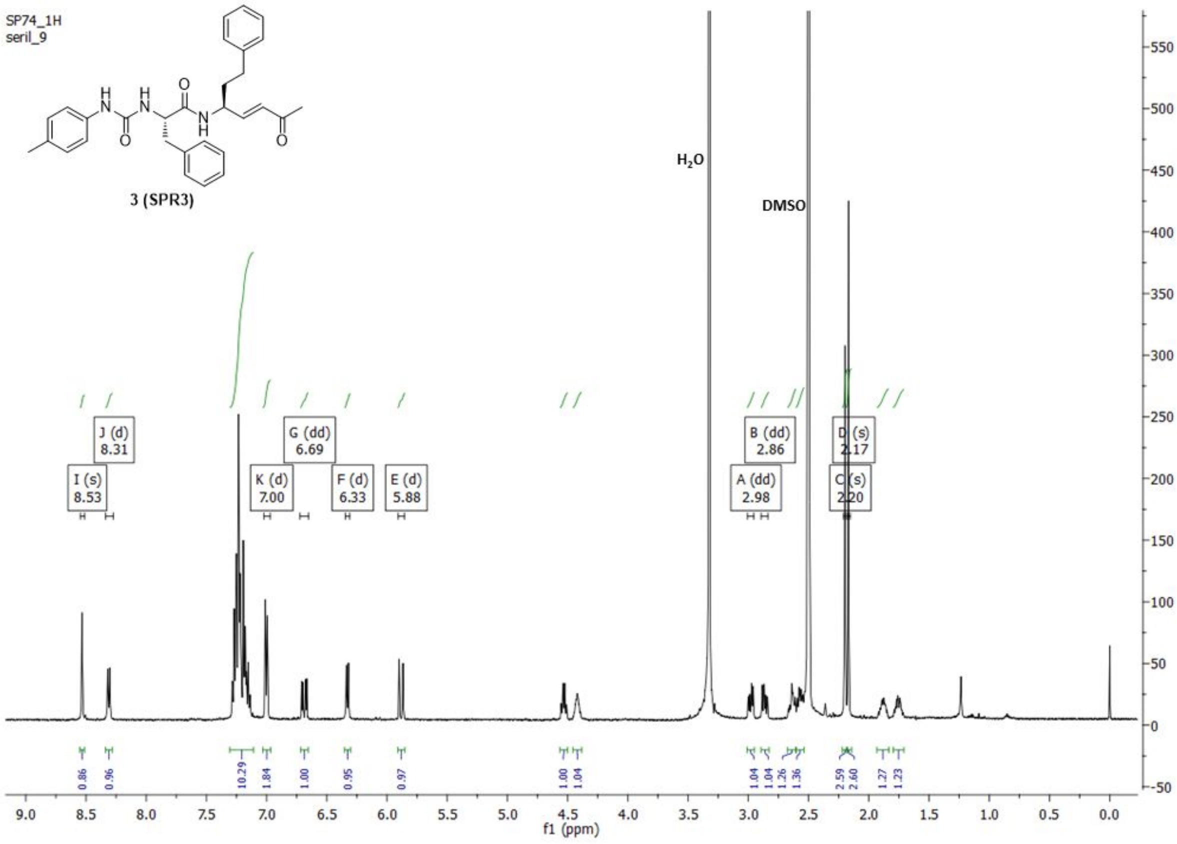

Figure S4. ^{1}H NMR of compound 3.

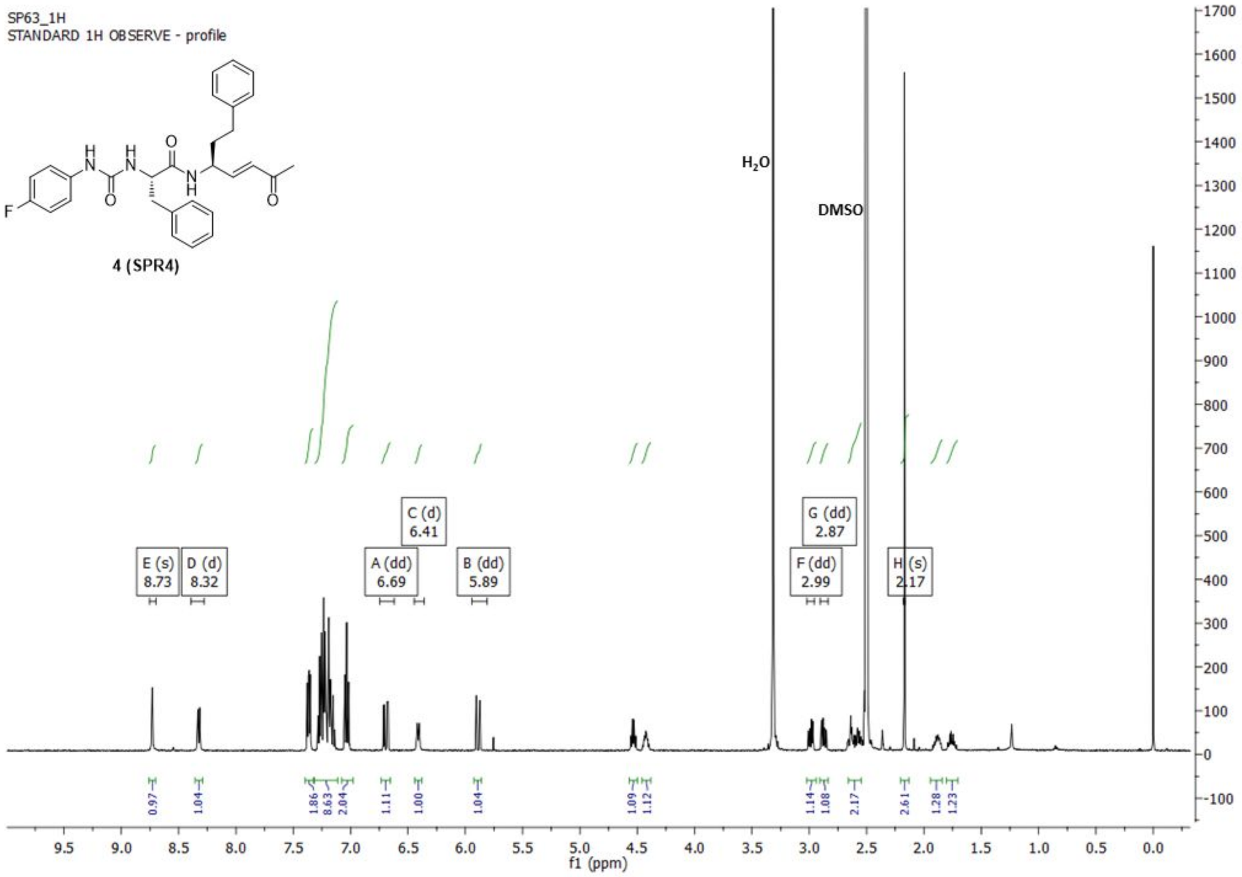

Figure S5. ¹H NMR of compound 4.

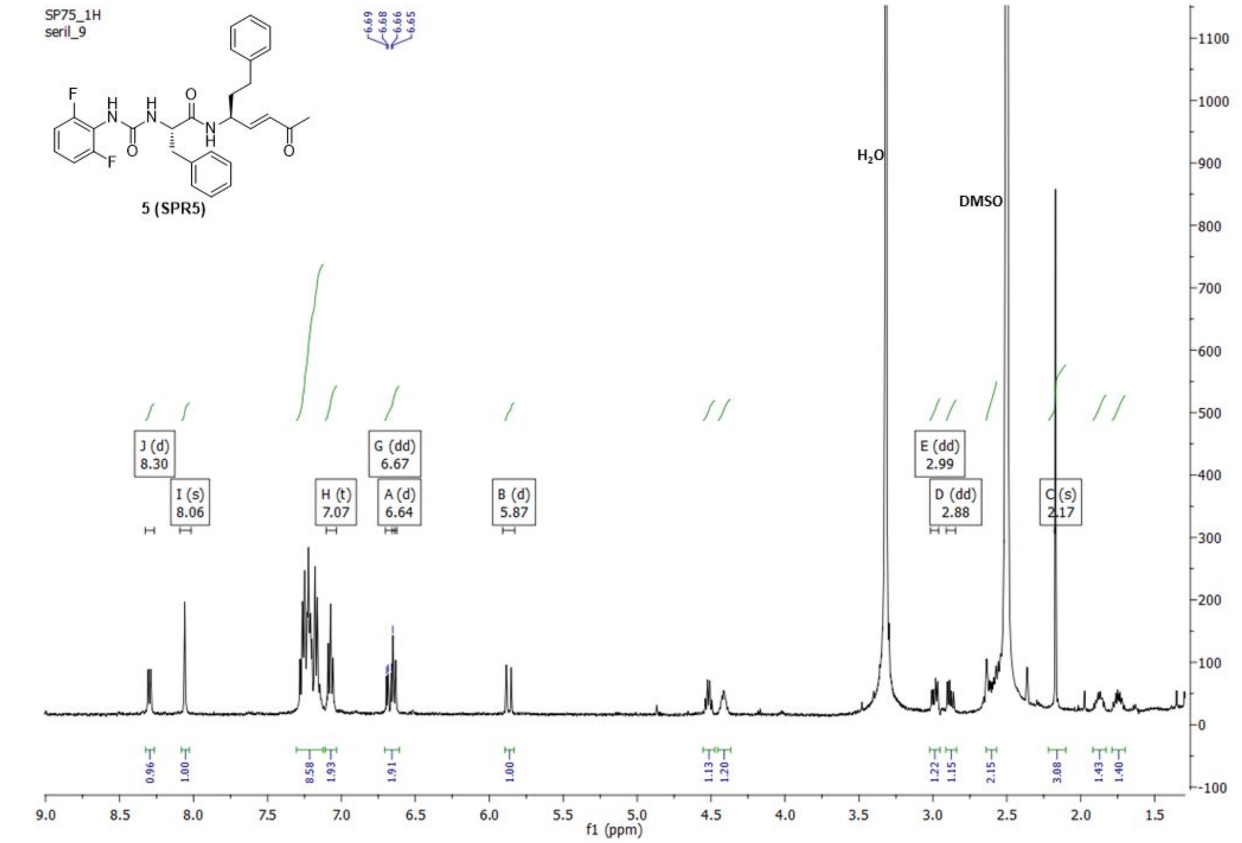

Figure S6. ¹H NMR of compound 5

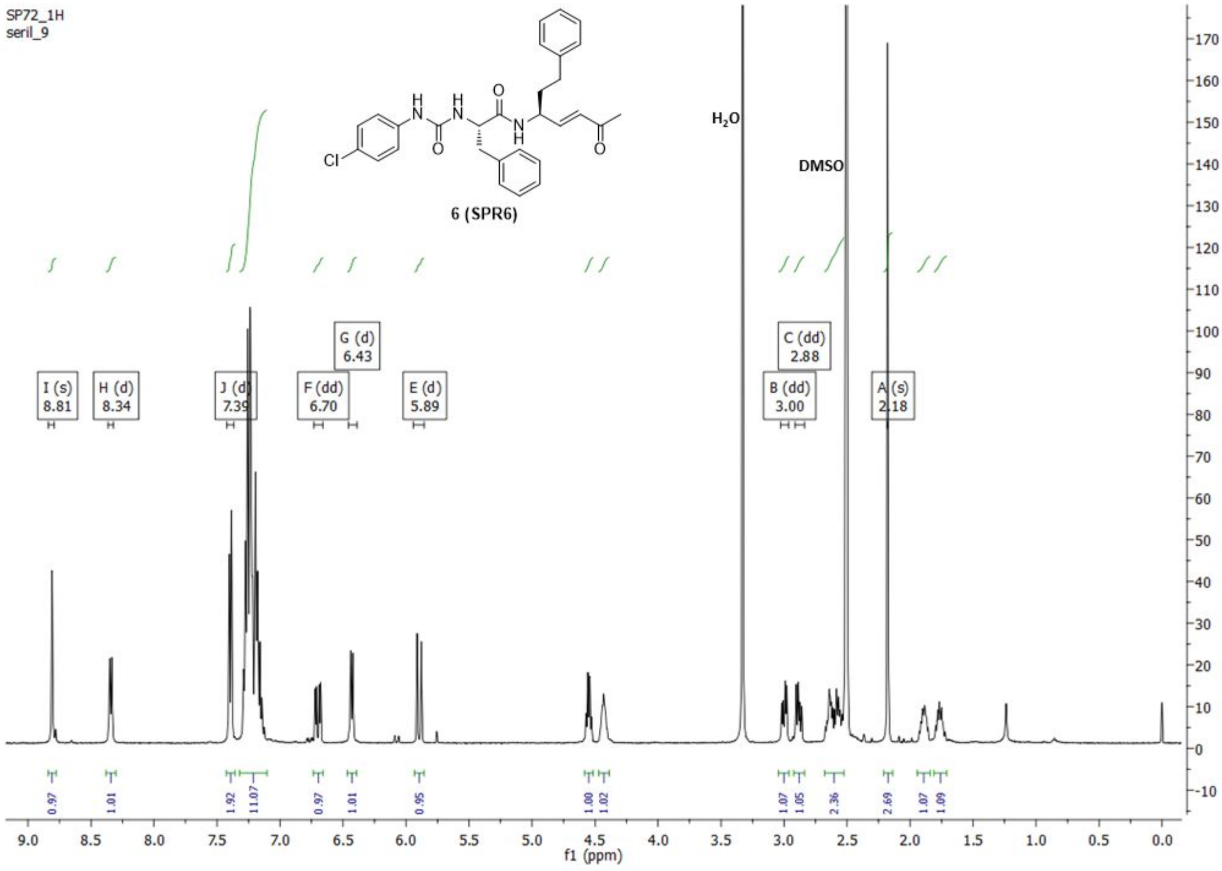

Figure S7. ^{1}H NMR of compound 6.

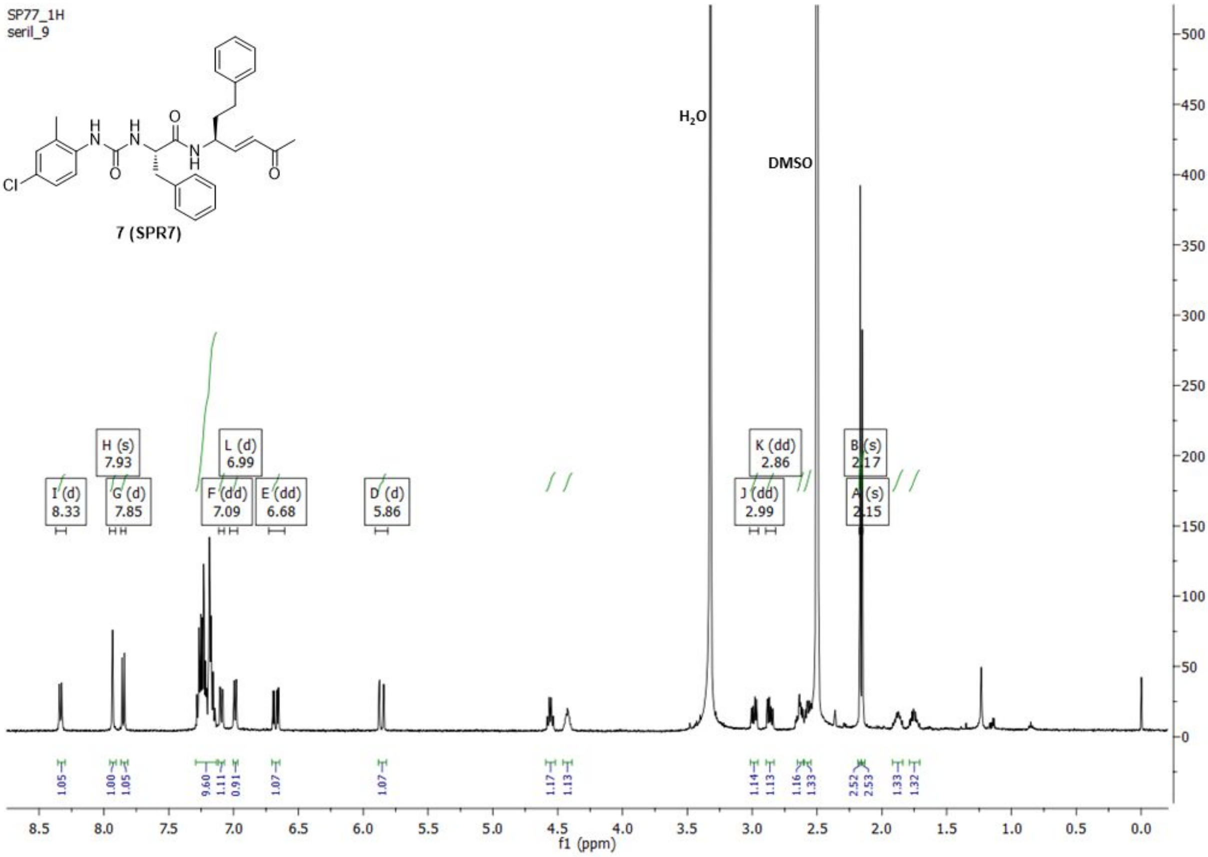

Figure S8. ^{1}H NMR of compound 7.

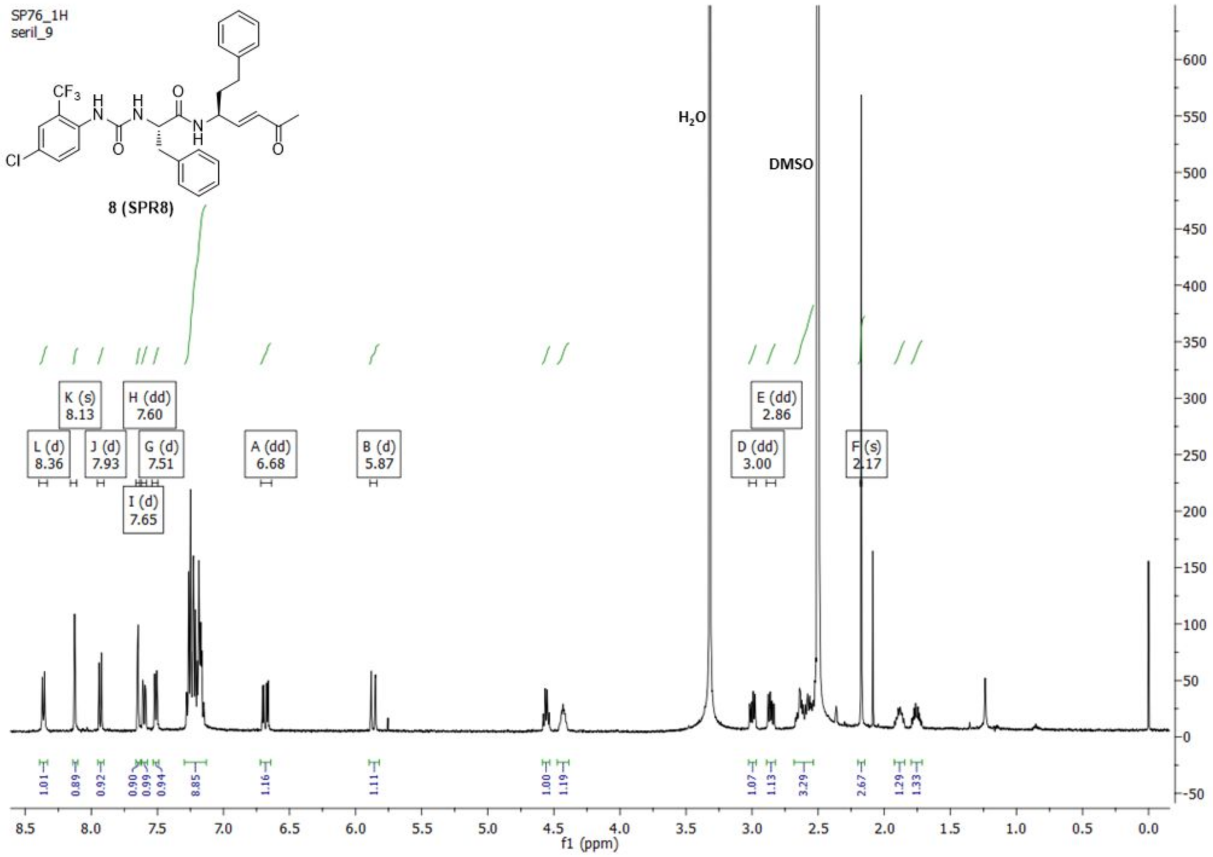

Figure S9. ^{1}H NMR of compound 8.

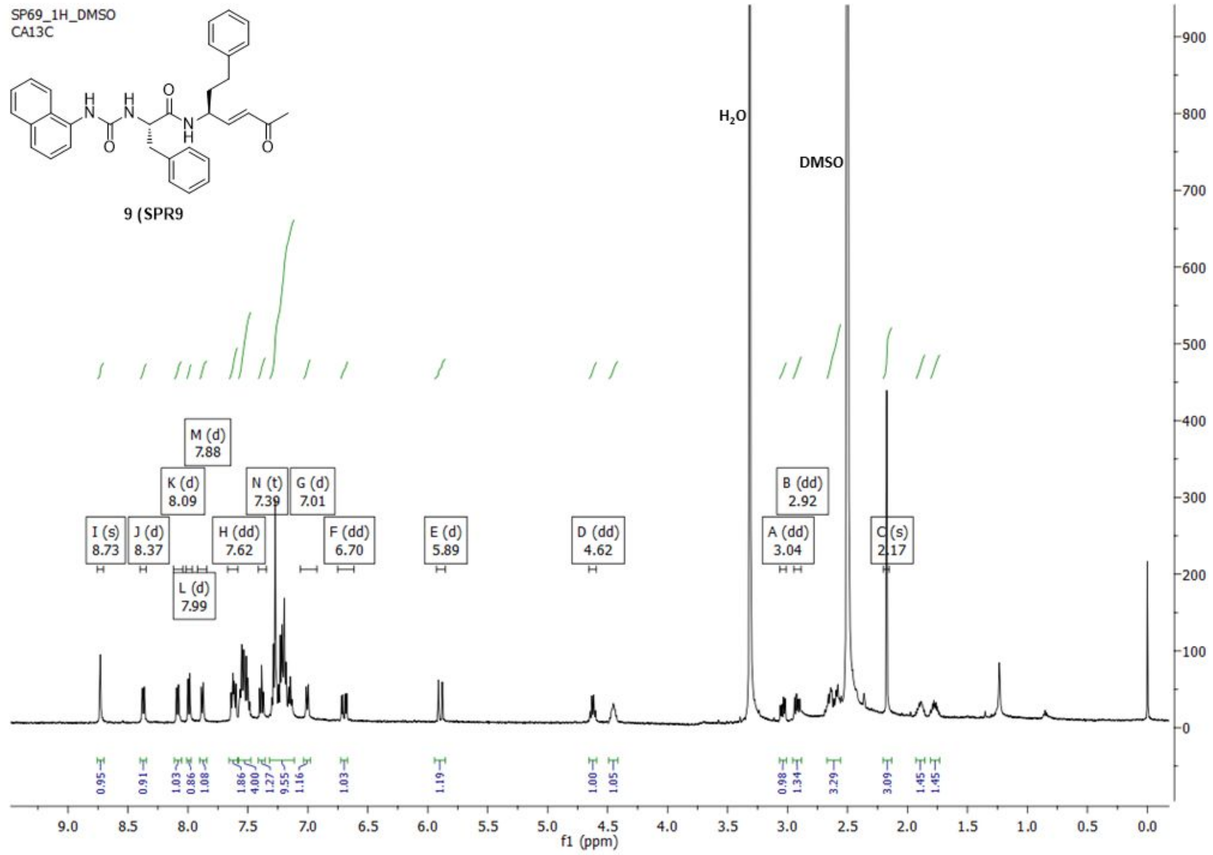

Figure S10. ^{1}H NMR of compound 9.

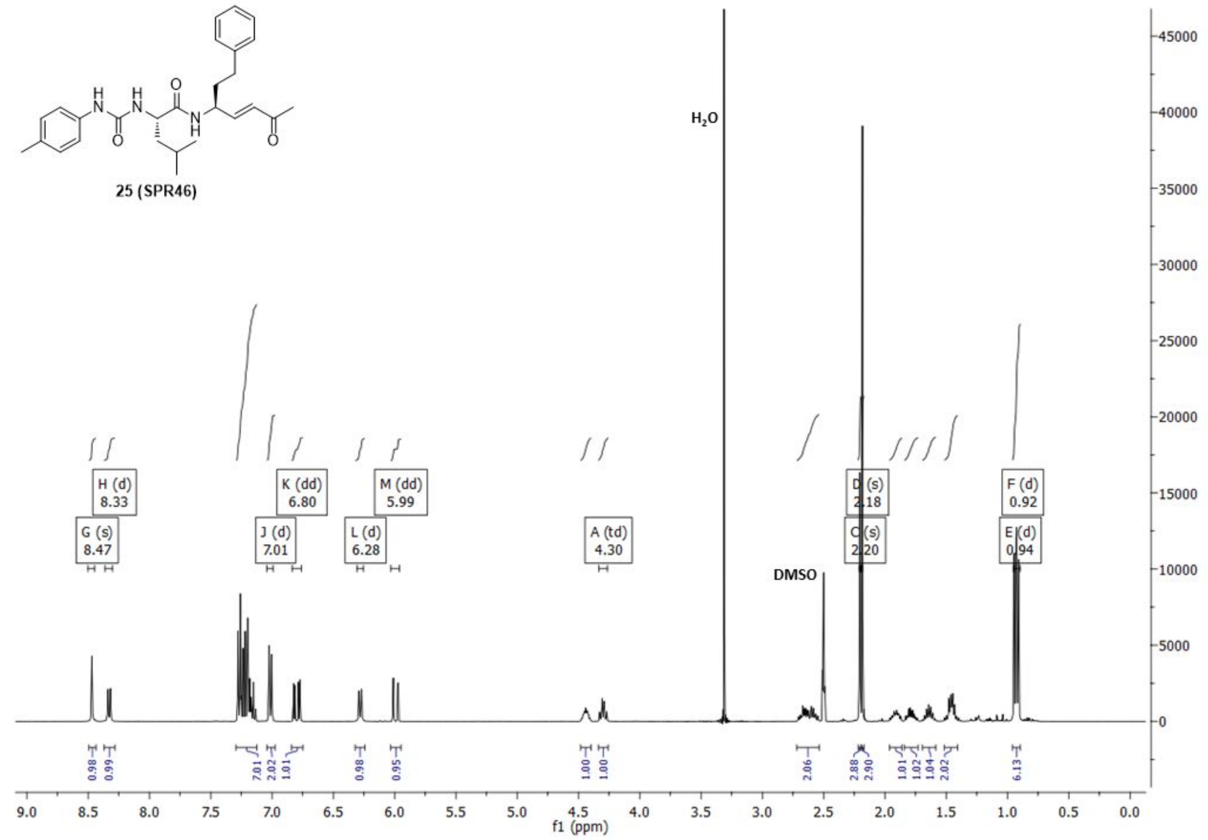

Figure S11. ^{1}H NMR of compound 25.

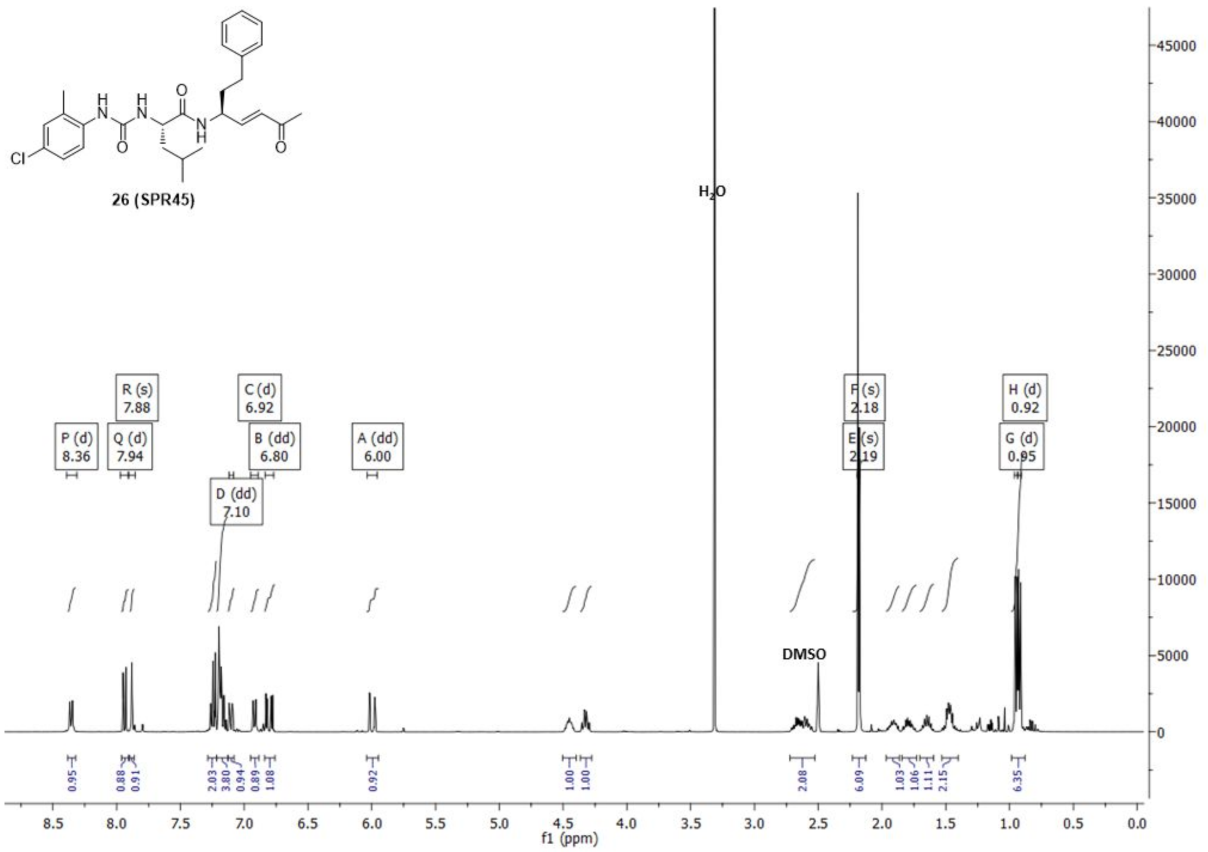

Figure S12. ^{1}H NMR of compound 26.

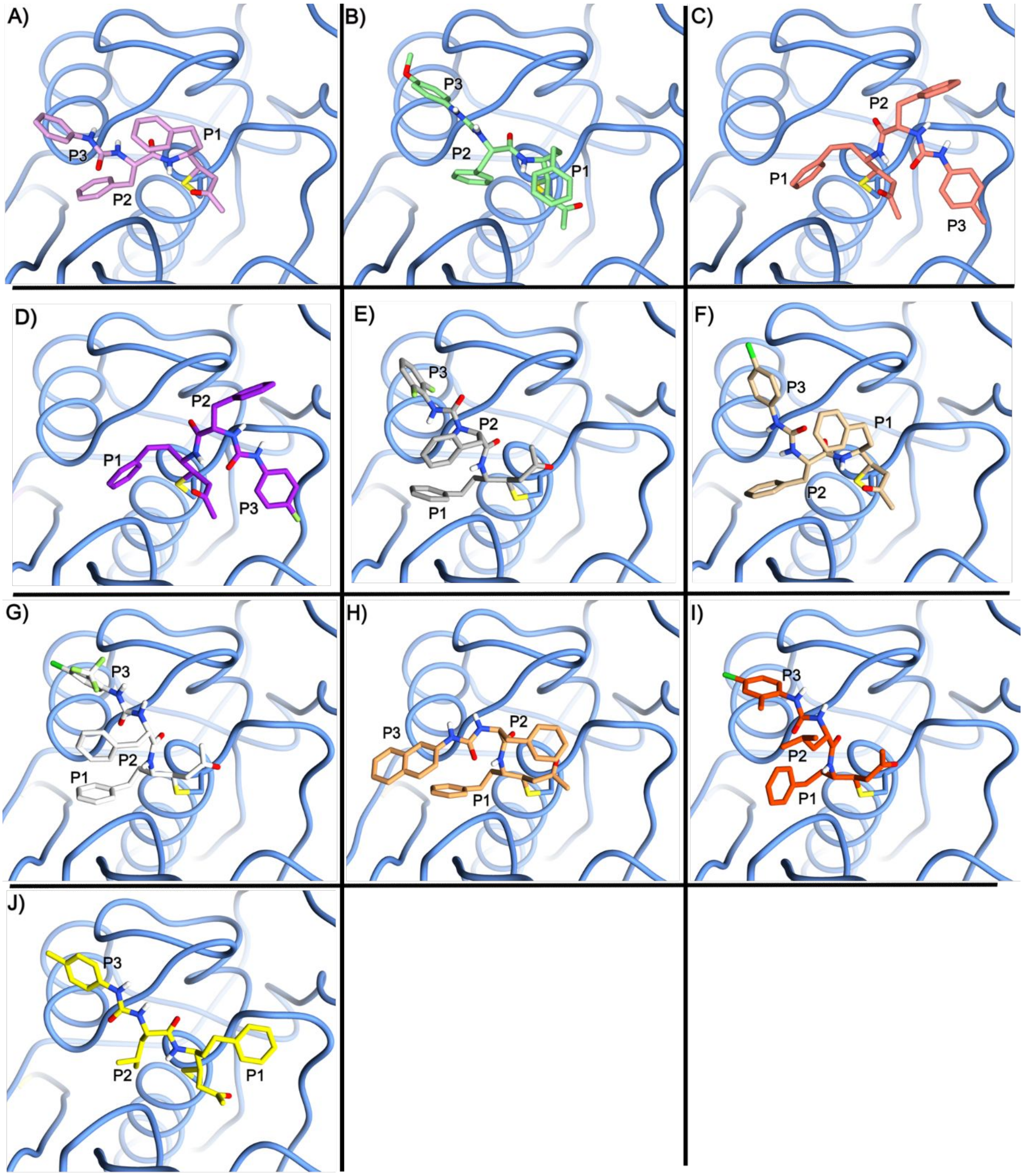

Figure S13. All the panels represent various molecules such as (A=**1**, B=**2**, C=**3**, D=**4**, E=**5**, F=**6**, G=**8**, H=**9**, I=**25**, J=**26**) having the P1, P2, P3 sites docked at the various sites of the rhodesain enzyme. The enzyme is depicted in blue ribbons and sticks. P1, P2, P3 sites are labelled. The images are rendered using UCSF Chimera.

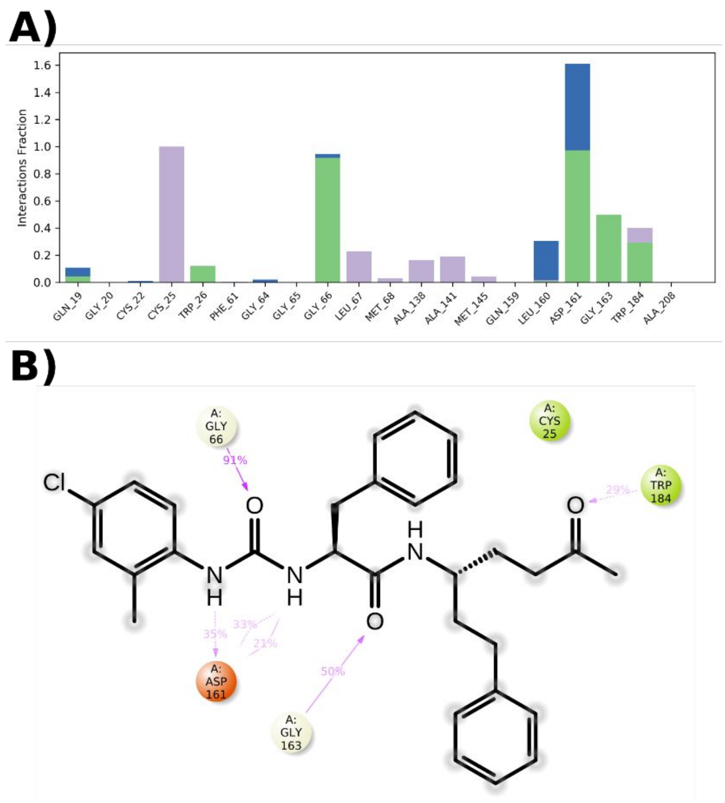

Figure S14. **(A)** Histogram representation of interaction occurrence between **7**/rhodesain throughout the simulation categorized into various water bridges, hydrogen bonds, hydrophobic and ionic bonds. **(B)** Schematic representation in 2D of the **7**/rhodesain interactions with the protein residues. Interactions that occur for more than 20% of the simulation time in the trajectory (0.00 through 100.00 ns) are shown.

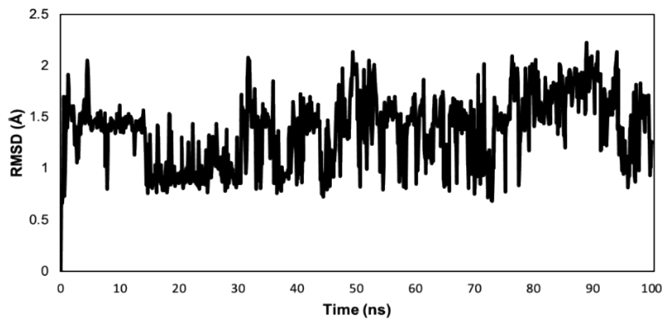

Figure S15. L-RMSD (Å) plot over time (ns) for compound **7** bound to rhodesain.

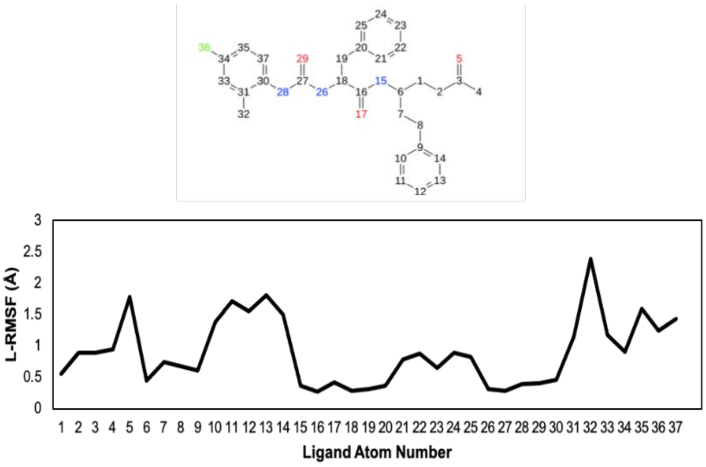

Figure S16. L-RMSF plot of compound **7** bound to rhodesain broken down by atom.

Table S1. Biological evaluation towards hCatL.

Cmp	k_{inact} (min^{-1})	K_i (nM)	k_{2nd} (x 10^{3}M^{-1} min^{-1})
**1**	0.0006 ± 0.0001	0.11 ± 0.01	5920 ± 150
**2**	0.0008 ± 0.0002	81.9 ± 1.06	9 ± 1
**3**	0.00045 ± 0.00005	1.32 ± 0.25	360 ± 106
**4**	0.0006 ± 0.0001	0.10 ± 0.01	5880 ± 120
**5**	0.0007 ± 0.0001	2.14 ± 0.25	326 ± 8
**6**	0.00075 ± 0.00005	3.11 ± 1.63	321 ± 152
**7**	0.00055 ± 0.00005	0.18 ± 0.03	3110 ± 160
**8**	0.00055 ± 0.00005	2.27 ± 0.43	256 ± 71
**9**	0.00065 ± 0.00005	0.051 ± 0.001	12820 ± 1050
**25**	0.00135 ± 0.00005	0.64 ± 0.02	2120 ± 6
**26**	0.00085 ± 0.00005	0.07 ± 0.03	14240 ± 5410
